# SAXS studies of the thermally-induced fusion of diblock copolymer spheres: formation of hybrid nanoparticles of intermediate size and shape†

**DOI:** 10.1039/d0sc00569j

**Published:** 2020-03-27

**Authors:** E. J. Cornel, P. S. O'Hora, T. Smith, D. J. Growney, O. O. Mykhaylyk, S. P. Armes

**Affiliations:** Department of Chemistry, University of Sheffield Dainton Building, Brook Hill Sheffield South Yorkshire S3 7HF UK o.mykhaylyk@sheffield.ac.uk s.p.armes@sheffield.ac.uk; Lubrizol Ltd Nether Lane, Hazelwood Derbyshire DE56 4AN UK

## Abstract

Dilute dispersions of poly(lauryl methacrylate)–poly(benzyl methacrylate) (PLMA–PBzMA) diblock copolymer spheres (a.k.a. micelles) of differing mean particle diameter were mixed and thermally annealed at 150 °C to produce spherical nanoparticles of intermediate size. The two initial dispersions were prepared *via* reversible addition–fragmentation chain transfer (RAFT) dispersion polymerization of benzyl methacrylate in *n*-dodecane at 90 °C. Systematic variation of the mean degree of polymerization of the core-forming PBzMA block enabled control over the mean particle diameter: small-angle X-ray scattering (SAXS) analysis indicated that PLMA_39_–PBzMA_97_ and PLMA_39_–PBzMA_294_ formed well-defined, non-interacting spheres at 25 °C with core diameters of 21 ± 2 nm and 48 ± 5 nm, respectively. When heated separately, both types of nanoparticles regained their original dimensions during a 25–150–25 °C thermal cycle. However, the cores of the smaller nanoparticles became appreciably solvated when annealed at 150 °C, whereas the larger nanoparticles remained virtually non-solvated at this temperature. Moreover, heating caused a significant reduction in mean aggregation number for the PLMA_39_–PBzMA_97_ nanoparticles, suggesting their partial dissociation at 150 °C. Binary mixtures of PLMA_39_–PBzMA_97_ and PLMA_39_–PBzMA_294_ nanoparticles were then studied over a wide range of compositions. For example, annealing a 1.0% w/w equivolume binary mixture led to the formation of a single population of spheres of intermediate mean diameter (36 ± 4 nm). Thus we hypothesize that the individual PLMA_39_–PBzMA_97_ chains interact with the larger PLMA_39_–PBzMA_294_ nanoparticles to form the hybrid nanoparticles. Time-resolved SAXS studies confirm that the evolution in copolymer morphology occurs on relatively short time scales (within 20 min at 150 °C) and involves weakly anisotropic intermediate species. Moreover, weakly anisotropic nanoparticles can be obtained as a final copolymer morphology over a restricted range of compositions (*e.g.* for PLMA_39_–PBzMA_97_ volume fractions of 0.20–0.35) when heating dilute dispersions of such binary nanoparticle mixtures up to 150 °C. A mechanism involving both chain expulsion/insertion and micelle fusion/fission is proposed to account for these unexpected observations.

## Abstract 2

Dilute dispersions of poly(lauryl methacrylate)-poly(benzyl methacrylate) diblock copolymer spheres of differing mean diameter are mixed and thermally annealed at 150 °C to produce either spherical or non-spherical nanoparticles of intermediate size.

## Graphical abstract

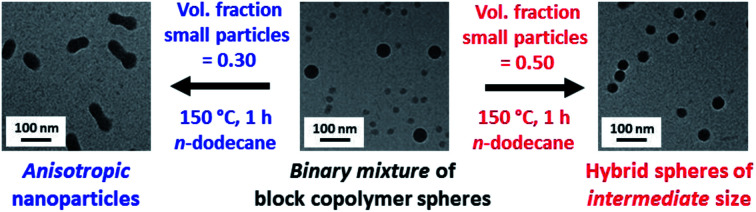

## Introduction

Block copolymer self-assembly has underpinned numerous remarkable advances in the field of materials science over the past two decades or so.^1^ For example, self-assembly in the solid state has led to the development of block copolymer nanolithography for information storage and nanofiltration.^2–5^ Similarly, self-assembly in solution has led to potential applications in microfluidic devices,^6^ automobile lubricants,^7^ viscosity modifiers,^8^ stem cell storage media^9^ and drug delivery.^10^

The first example of well-defined diblock copolymers were prepared by anionic polymerization.^11^ This living polymerization technique allows the synthesis of copolymer chains with very narrow molecular weight distributions but it is extremely sensitive to protic impurities and is applicable to only a narrow range of vinyl monomers. Fortunately, developments in the field of controlled radical polymerization, particularly reversible addition–fragmentation chain transfer (RAFT) polymerization, enable the preparation of many functional block copolymers under much less demanding reaction conditions.^12–19^

Traditionally, block copolymer self-assembly in solution has been performed *via* post-polymerization processing using a solvent-switch method.^1^ A much more convenient approach for the preparation of block copolymer nanoparticles involves RAFT-mediated polymerization-induced self-assembly (PISA).^17,20–28^ In essence, PISA involves chain extension of a soluble precursor block with a second insoluble block. Block copolymer self-assembly occurs *in situ* once the growing latter block reaches a critical mean degree of polymerization (DP). Polymerization continues thereafter within monomer-swollen nanoparticles. The high local monomer concentration leads to a rate acceleration while the unreacted monomer acts as a processing aid (or co-solvent). Given a sufficiently short steric stabilizer block, either spheres, worms/cylinders or vesicles obtained depending on the relative volume fractions of each block. In contrast, longer steric stabilizer blocks invariably lead to kinetically-trapped spheres, because sphere–sphere fusion is impeded.^20–22^

It is well-known that thermal annealing can lead to the exchange of diblock copolymer chains between nanoparticles in polar^29–35^ and non-polar media, as shown by both Lodge and co-workers and Growney *et al.*^36–43^ Two mechanisms have been suggested for this phenomenon: (i) a chain expulsion/insertion mechanism and (ii) a micelle fusion/fission mechanism.^41,44–48^ Theoretical studies by Halperin^45,46^ and experimental observations made by Lund and co-workers^31,35^ and Lodge and co-workers^36–39,41,49,50^ suggest that the former mechanism is much more likely for diblock copolymer micelles (a.k.a. sterically-stabilized nanoparticles). On the other hand, Armes and co-workers have suggested that particle–particle fusion is likely to play an important role during certain PISA syntheses, for which unreacted monomer plays an important processing role as a co-solvent. Indeed, such a fusion mechanism seems to be the most likely explanation for the *in situ* self-assembly of highly anisotropic diblock copolymer worms, whose formation is favored at higher copolymer concentrations.^21,22,51–53^ In principle, chain expulsion/insertion and micelle fusion/fission could each play important roles during RAFT PISA.

Herein we examine copolymer exchange for various binary mixtures of dilute copolymer dispersions comprising poly(lauryl methacrylate)–poly(benzyl methacrylate) (PLMA_39_–PBzMA_*x*_) spherical nanoparticles with mean core diameters of 21 ± 2 nm and 48 ± 5 nm, respectively. These kinetically-trapped spheres were prepared in *n*-dodecane *via* RAFT-mediated PISA using a previously reported protocol;^22,53^ the core-forming PBzMA_*x*_ block DP (*x*) was either 97 or 294. Variable temperature small-angle X-ray scattering (SAXS) studies provide valuable insights regarding the behavior of these nanoparticles when annealed separately at 150 °C with regard to their degree of core solvation and change in aggregation number. Annealing binary mixtures of this pair of nanoparticles over a wide range of relative volume fractions leads to the formation of a series of new hybrid nanoparticles. Time-resolved SAXS studies are used to examine the time scale for this hybridization process and also to examine its mechanism. This powerful characterization technique provides compelling evidence for the presence of weakly anisotropic intermediate species during the *in situ* evolution in copolymer morphology. Under certain conditions, weakly anisotropic nanoparticles can also be obtained as the final copolymer morphology from such hybridization experiments.

## Results and discussion

### Preparation and characterization of PLMA–PBzMA spheres in *n*-dodecane

**Scheme 1:**
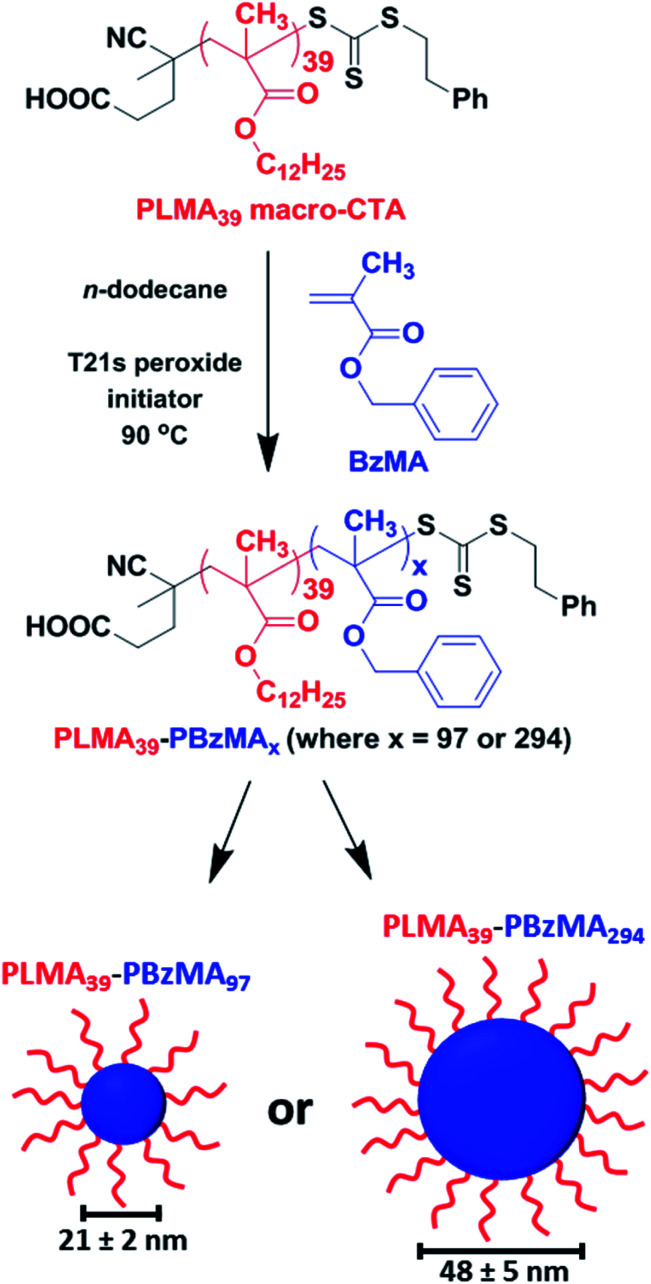
A PLMA_39_ precursor block is chain-extended with BzMA monomer in *n*-dodecane *via* RAFT-mediated PISA to form sterically-stabilized spherical nanoparticles. SAXS analysis (see Fig. S3, Tables S2 and S3 in the ESI†) indicated that final PBzMA DPs of 97 and 294 produced mean core diameters of 21 ± 2 nm and 48 ± 5 nm, respectively.

Well-defined PLMA_39_–PBzMA_*x*_ spherical nanoparticles (where *x* is either 97 or 294) were prepared at 20% w/w solids in *n*-dodecane using a well-established RAFT-mediated PISA protocol.^22,53^ A PLMA_39_ precursor was chain-extended with BzMA monomer, with micellar nucleation occurring once a critical PBzMA core DP was attained (Scheme 1). High BzMA monomer conversions (>97%) were achieved in both cases according to ^1^H NMR spectroscopy analysis of the crude reaction mixtures, after dilution with sufficient CDCl_3_ to ensure nanoparticle dissolution (Fig. S1 and eqn (S1) in the ESI†). Both types of diblock copolymer nanoparticles were dissolved in THF prior to GPC analysis (Fig. S2†). Relatively narrow unimodal molecular weight distributions were obtained (*M*_w_/*M*_n_ ≤ 1.20), suggesting pseudo-living character for this RAFT dispersion polymerization. A clear shift to lower retention time for each diblock copolymer compared to that for the PLMA_39_ precursor indicated a high blocking efficiency in each case. For closely-related PISA formulations, Fielding *et al.* reported that targeting higher PBzMA DPs simply led to progressively larger kinetically-trapped spherical nanoparticles when utilizing a sufficiently long PLMA stabilizer block.^22^ In the present study, SAXS patterns recorded at 25 °C for a 1.0% w/w dispersion of PLMA_39_–PBzMA_*x*_ nanoparticles could be fitted using a spherical micelle model^54,55^ (eqn (S2)–(S13) and Table S1†). This approach indicated spherical nanoparticle core diameters of 21 ± 2 nm and 48 ± 5 nm for the PLMA_39_–PBzMA_97_ and PLMA_39_–PBzMA_294_ spheres, respectively (Fig. S3, Tables S2 and S3†). The mean radius of gyration for the PLMA_39_ stabilizer chains was approximately 2.0 nm in both cases. This is consistent with the radius of gyration of 1.9 nm obtained by fitting the SAXS pattern recorded for a 1.0% w/w solution of PLMA39 homopolymer in *n*-dodecane (Fig. S4†) using the Debye function.^56^ SAXS pattern for the larger nanoparticles revealed an additional diffuse peak at *q* ∼ 0.8 nm^−1^ (Fig. S3B†). We account for this feature by including a Gaussian function in the data fit. This high *q* feature indicates a length scale of approximately 8 nm, which corresponds to twice the radius of gyration of the PBzMA chains (∼4 nm) within the nanoparticle cores.

### Variable temperature SAXS analysis of spherical nanoparticles

**Fig. 1:**
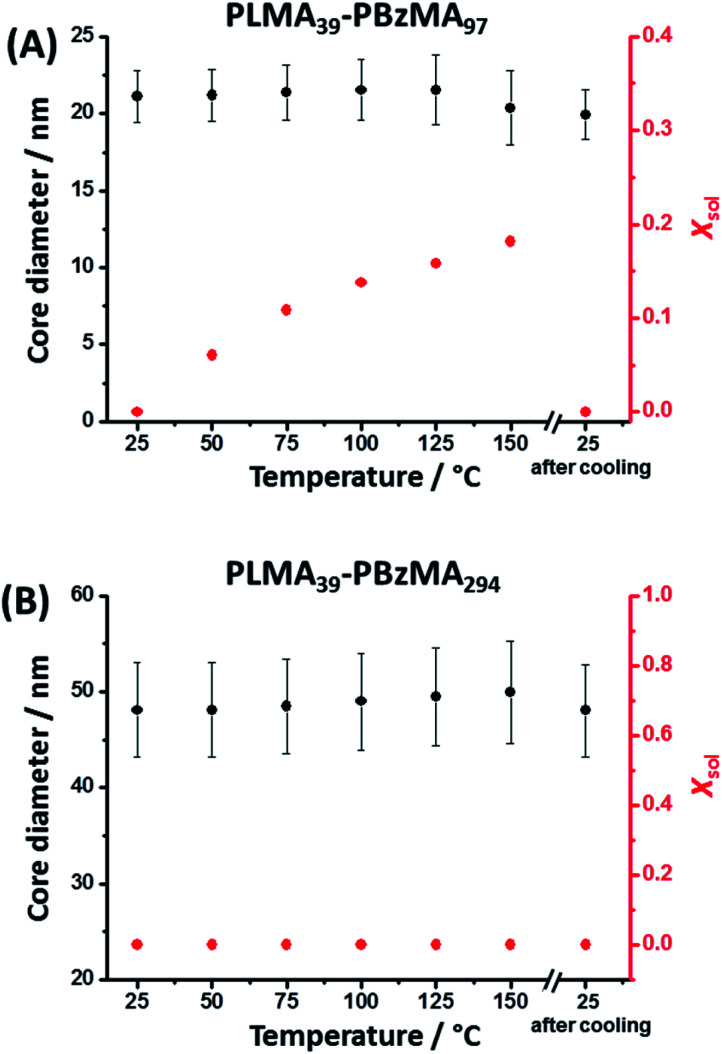
Mean nanoparticle core diameter and solvent volume fraction (*X*_sol_) determined for (A) PLMA_39_–PBzMA_97_ and (B) PLMA_39_–PBzMA_294_ spheres at various temperatures using SAXS analysis (Tables S2 and S3,† respectively). The core diameter remains approximately constant for both types of nanoparticles when heated up to 150 °C. However, progressively higher *X*_sol_ values are observed on heating PLMA_39_–PBzMA_97_ spheres. The original *X*_sol_ value of approximately zero is obtained for this system on returning to 25 °C. In contrast, PLMA_39_–PBzMA_294_ spheres exhibit no discernible change in *X*_sol_ on heating up to 150 °C. [*N.B.* Error bars correspond to the standard deviation in the mean nanoparticle diameter.]

It is well-known that copolymer chain exchange can occur between spherical diblock copolymer nanoparticles in non-polar media at elevated temperature. Moreover, such exchange is sensitive to both temperature and the DP of the core-forming block.^38,41^ It is also known that the cores of PLMA–PBzMA nano-objects become progressively more solvated with hot solvent at elevated temperature.^8,53^ It seems likely that the greater chain mobility associated with this solvent plasticization facilitates the chain expulsion/insertion mechanism, making the thermally-activated redistribution of copolymer chains more likely. Herein, variable temperature SAXS was used to study the degree of core solvation and integrity of PLMA_39_–PBzMA_97_ and PLMA_39_–PBzMA_294_ spheres at elevated temperature. In an initial series of experiments, dispersions of both types of nanoparticles were diluted in turn to 1.0% w/w using *n*-dodecane and scattering patterns were recorded in each case during a 25–150–25 °C thermal cycle (Fig. S3†). Heating PLMA_39_–PBzMA_97_ spheres alone led to a progressive change in the scattering pattern, which returned to its original form after cooling from 150 °C to 25 °C (Fig. S3A†). In contrast, heating the larger PLMA_39_–PBzMA_294_ spheres up to 150 °C produced almost no discernible change in the scattering pattern (Fig. S3B†). SAXS patterns recorded for the PLMA_39_–PBzMA_97_ and PLMA_39_–PBzMA_294_ spheres at various temperatures were fitted to a well-known spherical micelle model with an additional Debye function to account for a minor fraction of molecularly-dissolved PLMA_39_–PBzMA_*x*_ chains (see eqn (S2)–(S13) in the ESI†).^54–56^ Satisfactory data fits could be obtained for both types of nanoparticles by assuming that the change in mass density (which affects both the scattering length density and the individual block volumes) for the PLMA corona block and the PBzMA core-forming block was equal to that reported by Fetters and co-workers for poly(*n*-butyl methacrylate) and polystyrene, respectively (Table S1†).^57^ SAXS analysis indicated minimal change in mean diameter for both types of spherical nanoparticles on heating up to 150 °C (Fig. 1, Tables S2 and S3 in the ESI†). However, a progressive increase in the solvent volume fraction (*X*_sol_) within the nanoparticle cores was observed when heating the smaller PLMA_39_–PBzMA_97_ spheres. Moreover, a drastic reduction in nanoparticle concentration from 0.67 to 0.45% v/v was observed on heating to 150 °C. To account for this observation, the Debye function^56^ was used to include a population of molecularly-dissolved PLMA_39_–PBzMA_97_ chains in the data fit, such that this population becomes progressively larger at higher temperatures. Returning to 25 °C led to complete desolvation of the nanoparticle cores and the final data fit at this temperature indicated that only a minor population of PLMA_39_–PBzMA_97_ chains remained molecularly dissolved (Table S2†).

In contrast to the smaller nanoparticles, data fits obtained for the larger PLMA_39_–PBzMA_294_ spheres indicated minimal change in their mean diameter and degree of core solvation when thermally annealed at 150 °C. Moreover, the SAXS data fits indicate a rather more subtle increase in the relative concentration of the molecularly-dissolved PLMA_39_–PBzMA_294_ chains compared to that of the spherical nanoparticles (Table S3†). In summary, the above variable temperature SAXS experiments indicate that the cores of the larger spheres are much less plasticized by hot solvent compared to the smaller spheres, which means that the former nanoparticles are much less likely to undergo dissociation (*i.e.* expulsion of individual copolymer chains).

**Fig. 2:**
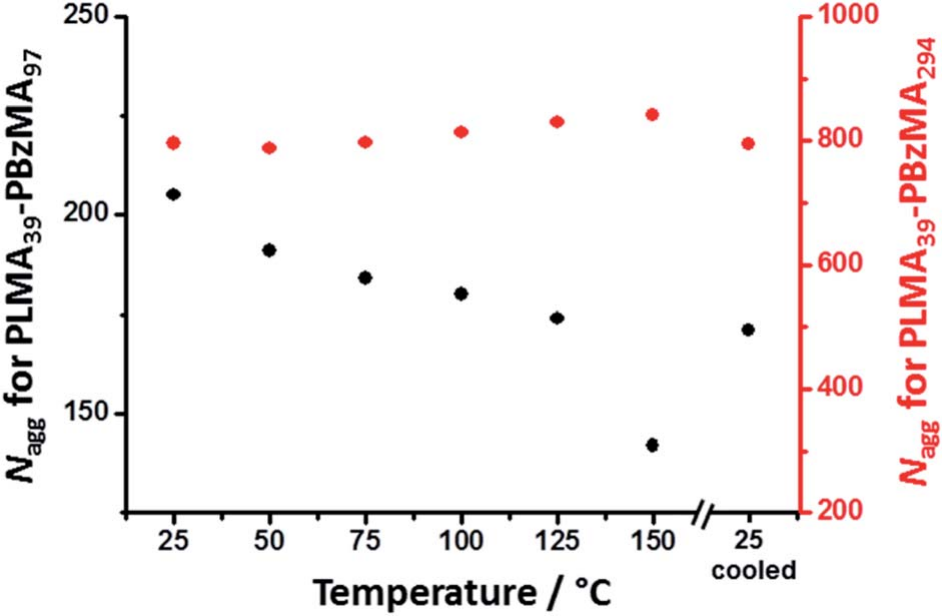
Temperature dependence for the mean aggregation number (*N*_agg_) determined by SAXS analysis of 1.0% w/w dispersions of (black) PLMA_39_–PBzMA_97_ and (red) PLMA_39_–PBzMA_294_ spheres at various temperatures. The former spheres exhibit a significant reduction in *N*_agg_ on heating, while the latter exhibit minimal change in aggregation number.

The mean core diameter for the highly solvated PLMA_39_–PBzMA_97_ nanoparticles at 150 °C is comparable to that of the same non-solvated nanoparticles at 25 °C. This implies a significant reduction in the volume-average aggregation number, *N*_agg_, (*i.e.* the mean number of copolymer chains per nanoparticle) at elevated temperature. This parameter was calculated using eqn (1):1
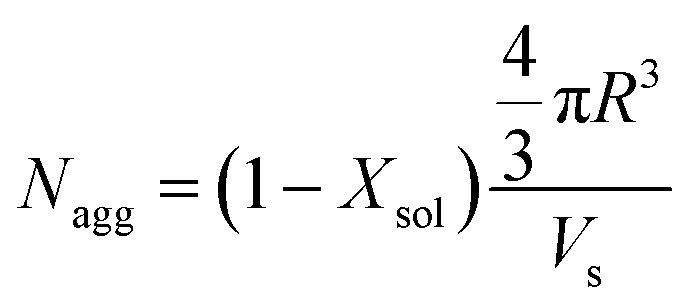
where *R* is the nanoparticle core radius and *V*_s_ is the volume occupied by a single PBzMA core-forming block. The calculated *N*_agg_ values are plotted against temperature for both PLMA_39_–PBzMA_97_ and PLMA_39_–PBzMA_294_ spheres (Fig. 2). The mean *N*_agg_ for the former spheres was significantly reduced from 205 at 25 °C to 142 at 150 °C, which is consistent with the formation of a secondary population of molecularly-dissolved diblock copolymer chains. Returning to 25 °C led to the formation of spheres with a slightly lower *N*_agg_ (171) than the original nanoparticles. It seems likely that annealing PLMA_39_–PBzMA_97_ spheres at 150 °C enables the diblock copolymer chains to rearrange to form spheres that lie closer to the thermodynamically-preferred size compared to the original spheres formed *via* RAFT-mediated PISA at 90 °C. Furthermore, these results suggest that a small fraction of molecularly-dissolved PLMA_39_–PBzMA_97_ chains remain dissolved after returning to 25 °C. In contrast, the minimal change in nanoparticle core diameter (along with the lack of solvation) observed for the larger PLMA_39_–PBzMA_294_ spheres during thermal annealing suggests an approximately constant *N*_agg_ during a 25–150–25 °C thermal cycle. In summary, SAXS analysis suggests that the smaller PLMA_39_–PBzMA_97_ spheres undergo dissociation to form molecularly-dissolved copolymer chains at 150 °C whereas the larger PLMA_39_–PBzMA_294_ spheres remain relatively stable under such conditions. It is perhaps noteworthy that this interpretation is consistent with the strong DP and diameter dependence for copolymer chain exchange reported by Lodge and co-workers.^38,41,58^

### Hybridization of PLMA_39_–PBzMA_97_ and PLMA_39_–PBzMA_294_ spheres

**Scheme 2:**
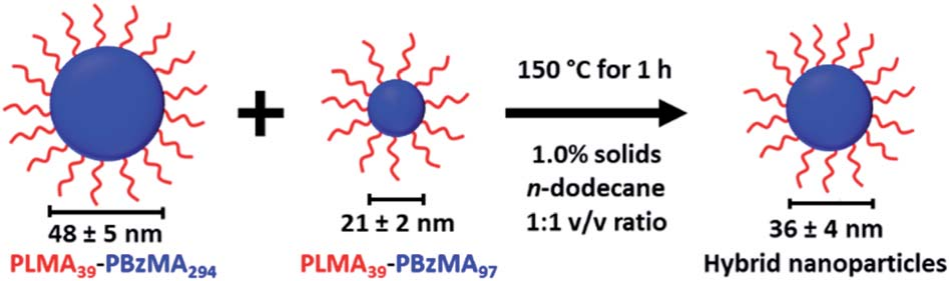
An equivolume binary mixture of PLMA_39_–PBzMA_97_ and PLMA_39_–PBzMA_294_ spheres at 1.0% w/w solids forms spheres of intermediate size when annealed at 150 °C for 1 h.

An equivolume binary mixture of PLMA_39_–PBzMA_97_ and PLMA_39_–PBzMA_294_ spheres at 1.0% w/w solids was heated up to 150 °C for 1 h to examine the possibility of thermally-activated exchange of copolymer chains between such nanoparticles. In initial experiments, thermal annealing of an equivolume binary mixture of these two types of nanoparticles leads to the formation of well-defined spherical nanoparticles of intermediate mean diameter (see Scheme 2).

**Fig. 3:**
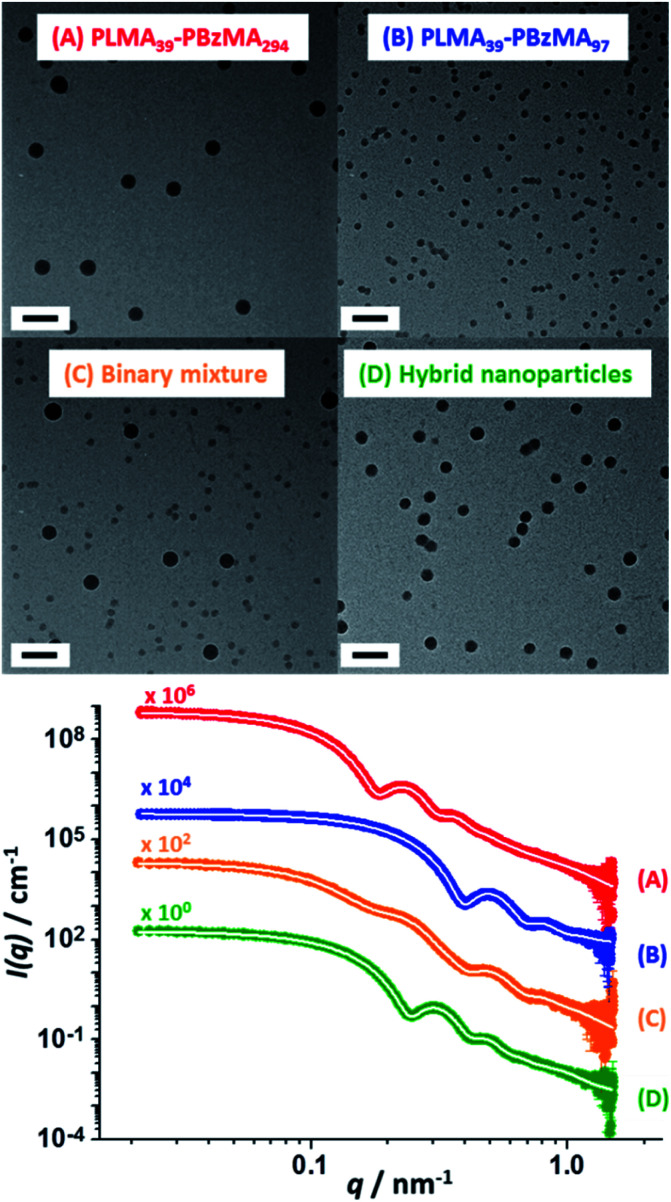
SAXS patterns (and corresponding TEM images) recorded at 25 °C for 1.0% w/w dispersions of: (A) PLMA_39_–PBzMA_294_ spheres and (B) PLMA_39_–PBzMA_97_ spheres; (C) a 1.0% w/w equivolume binary mixture of these two initial dispersions prior to thermal annealing and (D) the final hybrid nanoparticles formed after thermal annealing of the same binary mixture at 150 °C for 1 h. White traces indicate the best fits to the data obtained when using a spherical micelle model.^54–56^ Scale bars shown in TEM images correspond to 100 nm.

TEM analysis confirmed a well-defined spherical morphology for both types of nanoparticles prior to heating. As expected, the binary dispersion exhibited two distinct populations prior to thermal annealing. Interestingly, TEM analysis of this binary mixture after heating to 150 °C for 1 h indicated a *single* population of spherical nanoparticles exhibiting an *intermediate* mean particle diameter (Fig. 3). These TEM observations were supported by SAXS experiments performed at ambient temperature (Scheme 2 and Fig. 3). According to general scattering theorems, the scattering intensity in the low *q* region of the X-ray scattering pattern (*q* ∼ 0 Å^−1^) is proportional to the nanoparticle volume.^59^ Thus larger nanoparticles cause stronger X-ray scattering in this regime. Furthermore, the gradient of the pattern in this low *q* region is sensitive to the nanoparticle shape. For example, a zero gradient indicates the presence of isotropic spheres. In contrast, anisotropic nanoparticles exhibit a negative gradient in this low *q* region. Thus, platelets/disks or vesicles possess a gradient of −2, while long thin rods are characterized by a gradient of −1 (with less anisotropic rods typically possessing gradients ranging between zero and −1).^60^

The scattering patterns recorded for the two initial dispersions, their equivolume binary mixture and the final hybrid nanoparticles exhibited approximate zero gradients at low *q*, which indicates the presence of spherical non-interacting nanoparticles in each case. Moreover, the scattering pattern recorded for the initial binary mixture of nanoparticles could be satisfactorily fitted using the known size distribution for each component by simply varying the relative concentrations of the two types of nanoparticles (Table S4†). The SAXS pattern recorded for the annealed binary dispersion is consistent with the TEM images shown in Fig. 3: the local minimum in the scattering curve clearly falls between the two minima observed for the original large and small spherical nanoparticles, which confirms that hybrid spherical nanoparticles with an intermediate mean diameter are obtained after heat treatment. The scattering pattern recorded for these hybrid nanoparticles was fitted to the same spherical micelle model using a mean PBzMA core volume calculated from the known proportions of the two nanoparticle populations (Table S4†). Assuming complete entropic mixing, this fit to the scattering curve gave a mean volume-average core diameter of 36 ± 3 nm, which is consistent with the number-average core diameter of 34 ± 3 nm estimated from TEM analysis (Fig. 3D). This confirms that the dimensions of the hybrid nanoparticles lie between those of the two initial nanoparticle dispersions.

### *In situ* SAXS studies of the kinetics of nanoparticle hybridization

**Fig. 4:**
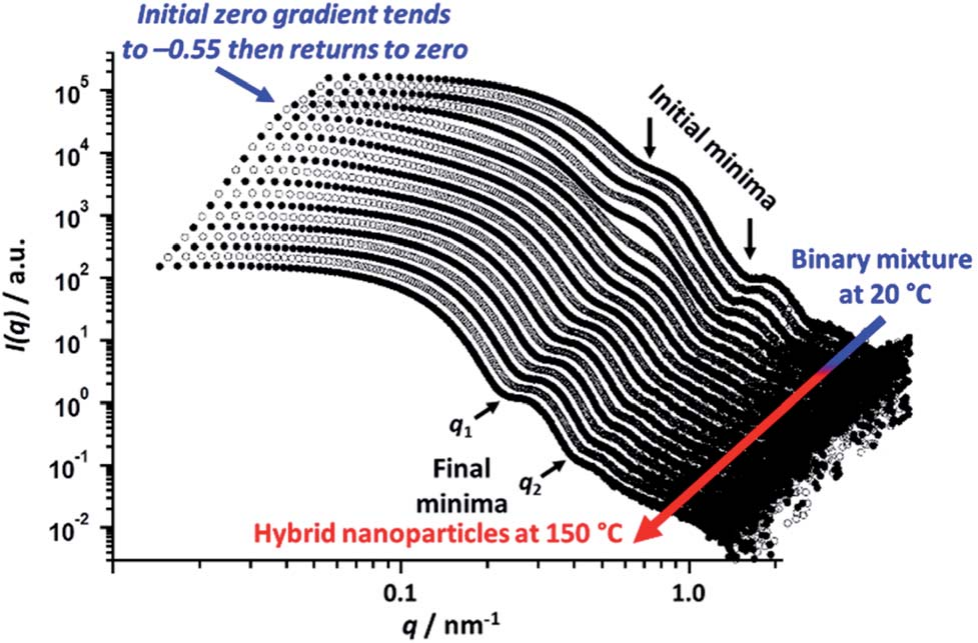
*In situ* SAXS patterns recorded when heating a 1.0% w/w nanoparticle dispersion comprising a 1 : 1 v/v binary mixture of 21 ± 2 nm PLMA_39_-PBzMA_97_ and 48 ± 5 nm PLMA_39_–PBzMA_294_ spheres up to 150 °C at a heating rate of 30 °C min^−1^. As expected, two minima are discernible in the initial pattern recorded for this binary mixture at 20 °C. However, the final scattering pattern obtained after annealing for 20 min at 150 °C shows two minima (denoted *q*_1_ and *q*_2_) that correspond to a single population of spherical nanoparticles of intermediate diameter (36 ± 4 nm).

To explore the nanoparticle hybridization mechanism, *in situ* SAXS measurements were performed while heating a 1.0% w/w dispersion comprising an equivolume binary mixture of PLMA_39_–PBzMA_97_ and PLMA_39_–PBzMA_294_ spheres up to 150 °C (Fig. 4). A scattering pattern for the initial binary mixture was recorded at 20 °C, with two distinct minima representing the bimodal nature of the initial nanoparticle dispersion (Fig. 3). After heating up to 150 °C at 30 °C min^−1^, the first scattering pattern recorded at this temperature also exhibited these two minima. However, maintaining this dilute binary dispersion at 150 °C produced a low *q* gradient of −0.55 within 7.0 min. Thereafter, a low *q* gradient of approximately zero was again observed (within a further 12.5 min). The final scattering pattern acquired after thermal annealing for 20 min at 150 °C exhibited two minima that correspond to a single population of spherical nanoparticles (Scheme 2). [*N.B.* For these two features, we find that *q*_1_*R* = 4.49 and *q*_2_*R* = 7.73, where *R* corresponds to the core radius of the final hybrid spherical nanoparticles, Fig. 4.] Thus *R* is calculated to be approximately 18 nm in each case, which indicates a mean particle diameter of ∼36 nm. In summary, these *in situ* SAXS studies strongly suggest that the transformation of the initial binary mixture of spheres (Fig. 3C) into a single population of spheres of intermediate diameter (Fig. 3D) involves weakly anisotropic transient species.

**Fig. 5:**
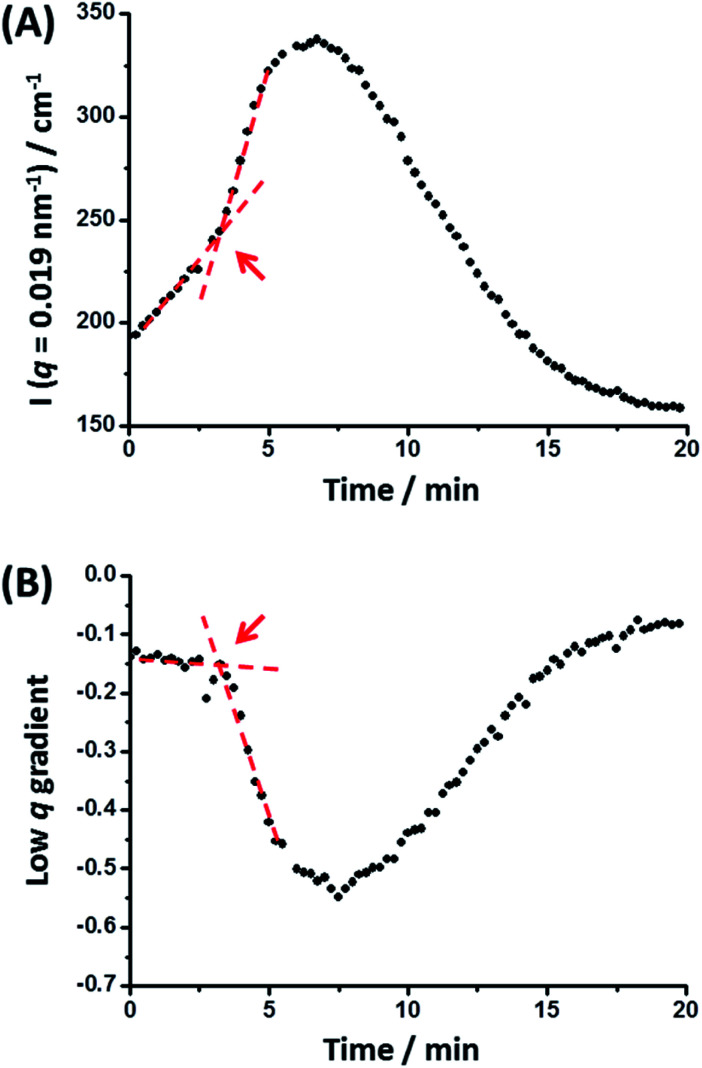
(A) Variation in scattered X-ray intensity, *I*(*q*), at an arbitrary *q* value of 0.019 nm^−1^ and (B) change in the low *q* gradient (for 0.019 nm^−1^ < *q* < 0.035 nm^−1^) over time recorded during an *in situ* SAXS experiment performed at 150 °C whereby spherical nanoparticles of intermediate diameter (36 ± 4 nm) are generated from a dilute 1 : 1 v/v binary mixture of spherical nanoparticles with initial volume-average diameters of 21 ± 2 nm and 48 ± 5 nm, respectively (see Fig. 4 caption for further experimental details). The red lines and arrows indicate that a significant change in both the low *q* gradient and the scattered X-ray intensity occurs after 3.2 min, which corresponds to approximately 116 °C.

The scattered X-ray intensity at an arbitrary *q* value of 0.019 nm^−1^ (Fig. 5A), and the low *q* gradient in the 0.019 nm^−1^ < *q* < 0.035 nm^−1^ interval (Fig. 5B) were plotted against time to further investigate the mechanism of formation of the final hybrid nanoparticles. Using a heating rate of 30 °C min^−1^, the final temperature of 150 °C was achieved within 4.3 min during these *in situ* SAXS experiments. Inspecting Fig. 5, both the scattered intensity and the low *q* gradient begin to change just below 150 °C, suggesting that nanoparticle hybridization has already commenced prior to the target temperature being attained. A pronounced maximum in scattered X-ray intensity is observed at around 7.0 min (Fig. 5A), which roughly corresponds to the formation of the most anisotropic transient species as judged by the change in the low *q* observed gradient (Fig. 5B). It should also be noted that the initial scattered X-ray intensity of 199 cm^−1^ is higher than the final intensity (159 cm^−1^) (Fig. 5A). This is understandable because the X-ray scattering at low *q* is proportional to the volume of the scattering objects. As a result, X-ray scattering from the original equivolume binary mixture of large and small nanoparticles should be dominated by the former population, which also scatter more strongly than the final spheres of intermediate size [this latter point is readily illustrated by simple calculation, *i.e.* (48^3^ + 21^3^)/2 > 36^3^].

The progressive change in the low *q* gradient during thermal annealing indicates the formation of weakly anisotropic species (mean aspect ratio = 2–3). This unexpected observation suggests that the change in copolymer morphology is unlikely to simply involve expulsion of PLMA_39_–PBzMA_97_ copolymer chains from the smaller nanoparticles and their subsequent insertion into the larger PLMA_39_–PBzMA_294_ nanoparticles. This is because such a copolymer chain exchange mechanism should simply reduce the diameter of the smaller nanoparticles while increasing that of the larger nanoparticles.^61^ Given that a spherical morphology has the lowest possible surface area per unit mass, it is difficult to envisage how such mass transport could result in the formation of non-isotropic nanoparticles. It seems much more likely that the transient anisotropic species are instead obtained *via* a fusion/fission process, despite the relatively low copolymer concentration (1.0% w/w) used for these thermal annealing experiments. However, given that the extent of core solvation and mean aggregation number strongly depend on the DP of the core-forming block (Fig. 1 and 2), relatively few of the longer PLMA_39_–PBzMA_294_ chains are expected to be expelled from the larger nanoparticles at 150 °C. Actually, the gradual increase in X-ray scattering intensity at *q* = 0.019 nm^−1^ and the low *q* gradient of approximately zero indicates that the *initial* nanoparticles become progressively larger at 150 °C while maintaining their spherical morphology (Fig. 5, red lines and arrows). Thus, this observation suggests that the expelled PLMA_39_–PBzMA_97_ chains are simply incorporated within the larger PLMA_39_–PBzMA_294_ spheres for the first 3.2 min.

However, a significant increase in X-ray scattering intensity is observed after 3.2 min, which is accompanied by an abrupt change in the low *q* gradient. These observations are consistent with the formation of larger, weakly anisotropic nanoparticles, *e.g.* dimers/trimers. The subsequent reduction in X-ray scattering intensity and return to a low *q* gradient of approximately zero indicate the gradual evolution of these transient species into the final isotropic spheres of intermediate core diameter. Presumably, this latter process is driven by the minimization of surface free energy (see below for a proposed mechanism).

### Effect of varying the relative volume fraction of small nanoparticles on copolymer morphology

**Fig. 6:**
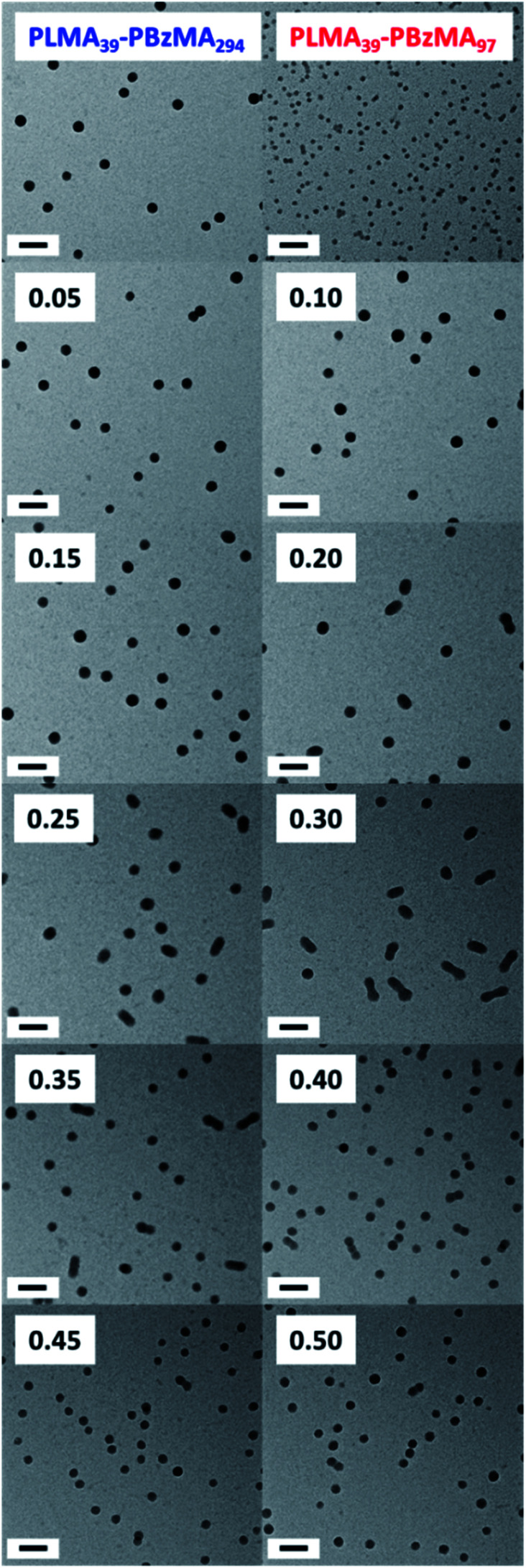
TEM images recorded for the series of hybrid nanoparticles formed after annealing 1.0% w/w binary mixtures of PLMA_39_–PBzMA_97_ and PLMA_39_–PBzMA_294_ spheres at 150 °C for 1 h. Caption labels denote the volume fraction of the smaller PLMA_39_–PBzMA_97_ spheres used in these experiments. The first two images correspond to the initial spherical nanoparticles annealed *individually* at 1.0% w/w solids in control experiments. All scale bars correspond to 100 nm. [*N.B.* The core diameters estimated from these TEM images are consistent with the corresponding SAXS studies, see Fig. 7].

Varying proportions of small PLMA_39_–PBzMA_97_ spheres were annealed with large PLMA_39_–PBzMA_294_ spheres in an attempt to produce the anisotropic intermediate species as a final hybrid copolymer morphology. These binary mixtures were prepared at various volumetric ratios at 20% w/w solids, then diluted to 1.0% w/w using *n*-dodecane and heated to 150 °C for 1 h. PLMA_39_–PBzMA_97_ volume fractions ranging from 0.05 to 0.50 were examined in these nanoparticle fusion experiments. Indeed, TEM analysis of the annealed dispersions revealed formation of the anticipated weakly anisotropic nanoparticles (mean aspect ratio = 2–3) when using PLMA_39_–PBzMA_97_ volume fractions of between 0.20 and 0.35 (Fig. 6). However, it is perhaps worth emphasizing that such species always co-exist with a variable population of spheres.

**Fig. 7:**
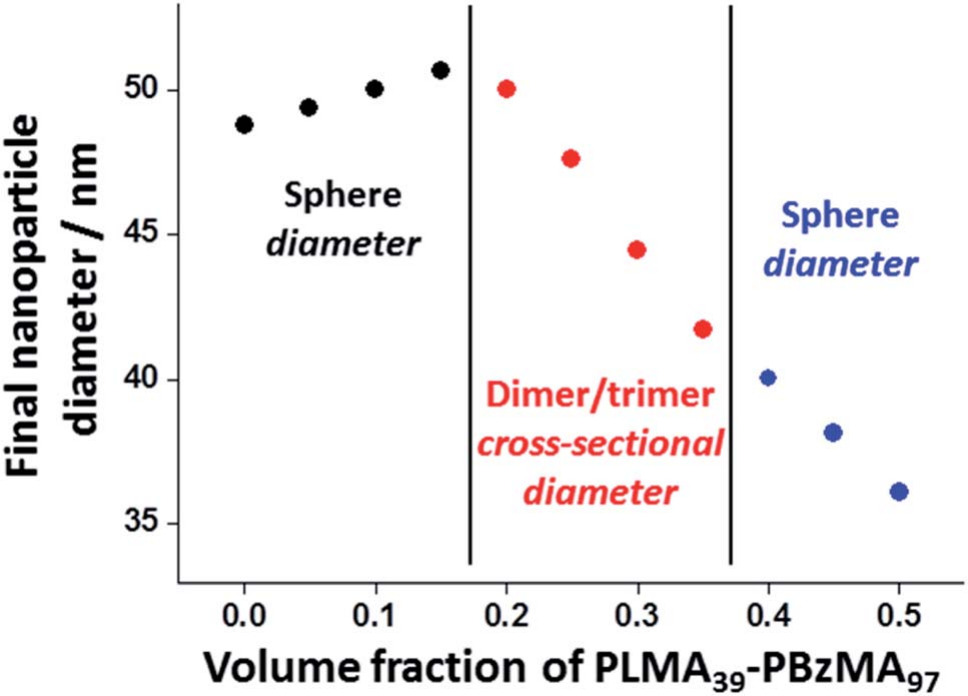
Change in nanoparticle dimensions (and morphology) observed when varying the volume fraction of PLMA_39_–PBzMA_97_ nanoparticles in thermal annealing experiments conducted at 150 °C using a 1.0% w/w binary mixture of PLMA_39_–PBzMA_97_ and PLMA_39_–PBzMA_294_ nanoparticles in *n*-dodecane (see TEM images in Fig. 6 and corresponding SAXS patterns in Fig. S5†). Hybrid nanoparticle dimensions (*i.e.* either sphere diameter or mean cross-sectional diameter of the dimers/trimers) were calculated from the *q* value for the first minima in the corresponding SAXS patterns (particle dimension = 4.49/*q*_min_, where the numerical factor corresponds to the first minimum for a spherical form factor).

Further insight regarding the formation of these kinetically stable hybrid nanoparticles was obtained by SAXS analysis (Fig. S5†). Scattering patterns for these dispersions were recorded at 1.0% w/w solids after thermal annealing at this concentration. The structure factor can be assumed to be unity for such dispersions and the approximate zero gradient observed at low *q* indicated a spherical morphology in most cases. However, a non-zero gradient was observed at low *q* when using PLMA_39_–PBzMA_97_ volume fractions of 0.20, 0.25, 0.30 or 0.35, indicating the presence of anisotropic nanoparticles. Moreover, these scattering patterns could not be fitted using a spherical micelle model (eqn (S2)–(S13) in the ESI) (Fig. S5†). These observations are consistent with TEM studies of these four thermally-annealed dispersions, for which a variable population of weakly anisotropic nanoparticles was observed at 20 °C (Fig. 6). These SAXS patterns were further analyzed by calculating nanoparticle dimensions (*i.e.* either the mean sphere diameter or a cross-sectional diameter for the dimer/trimer species) from the *q* value of the first minimum (particle dimension = 4.49/*q*_min_, where 4.49 corresponds to the first minimum for the spherical form factor), and plotting such sizes against the corresponding PLMA_39_–PBzMA_97_ volume fraction (Fig. 7). A modest monotonic increase in sphere diameter was observed for PLMA_39_–PBzMA_97_ volume fractions up to 0.15. At higher volume fractions (0.20–0.35), a population of weakly anisotropic transient particles is indicated by the gradual reduction in mean particle dimension, *i.e.* the effective cross-section of the dimers/trimers (Fig. 7). Finally, using a higher proportion of small PLMA_39_–PBzMA_97_ spheres (volume fraction = 0.40–0.50) leads to a gradual reduction in the mean sphere diameter. In this case, it appears that there is a sufficiently high concentration of PLMA_39_–PBzMA_97_ chains to generate a single population of spheres of intermediate size, as observed in Fig. 3 and 6.

### Proposed mechanism for nanoparticle hybridization

**Scheme 3:**
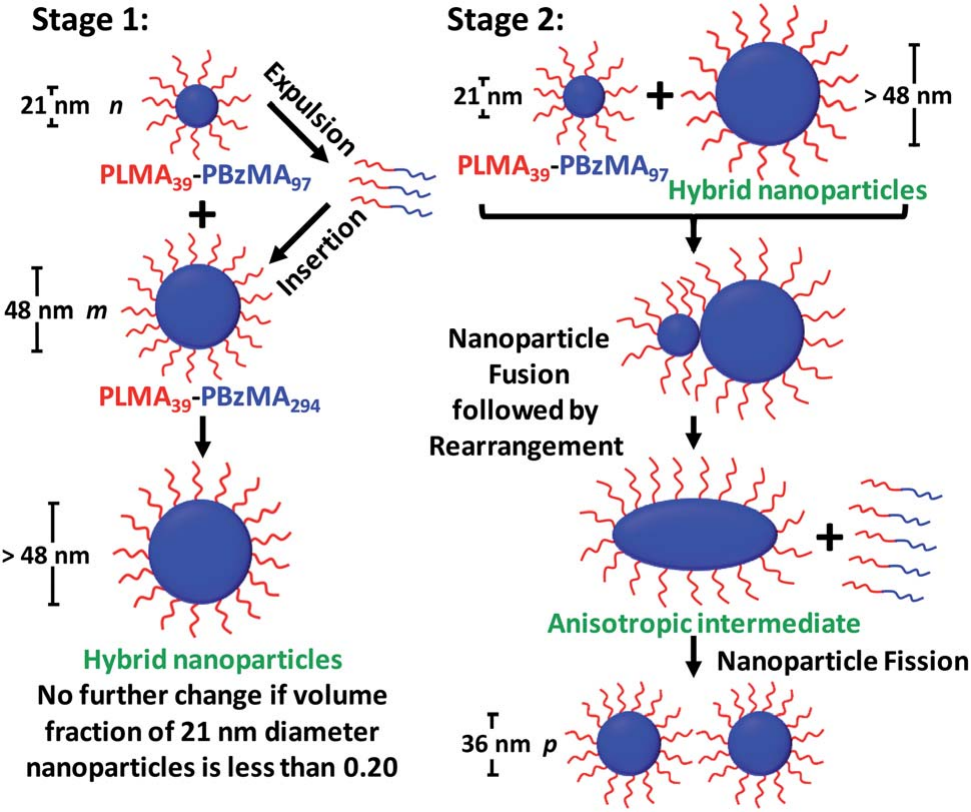
Schematic representation of the two-stage mechanism proposed for the changes in copolymer morphology that are observed during thermal annealing of a binary mixture of 21 ± 2 nm and 48 ± 5 nm diblock copolymer spheres at 150 °C. Here, the *n*, *m* and *p* values refer to the number density of each type of nanoparticle. In Stage 1, the smaller PLMA_39_–PBzMA_97_ spheres undergo partial dissociation to form copolymer chains, which then become incorporated into the larger spheres to produce hybrid spheres with a mean diameter greater than 48 nm. If the volume fraction of these smaller spheres is less than 0.20, this is the final copolymer morphology. However, using higher volume fractions of this component leads to Stage 2, whereby the 21 nm spheres undergo fusion with the larger hybrid spheres to form weakly anisotropic transient species. The latter then undergo fission – most likely mediated by incorporation of further PLMA_39_–PBzMA_97_ chains – to form spheres of intermediate size (*e.g.* 36 nm diameter). This mechanism is consistent with the SAXS data shown in Fig. 3–5 and 7 and the TEM images shown in Fig. 3 and 6.

Taking into consideration the TEM images and SAXS data discussed above, a tentative two-stage mechanism is proposed in Scheme 3. In Stage 1, individual copolymer chains are expelled from the PLMA_39_–PBzMA_97_ nanoparticles at 150 °C and are reinserted into the larger PLMA_39_–PBzMA_294_ nanoparticles, which barely undergo any copolymer exchange under such conditions (see Fig. 1 and 2). This causes the latter nanoparticles to grow in size, which simply leads to the formation of larger hybrid spheres provided that the volume fraction of the smaller PLMA_39_–PBzMA_97_ nanoparticles is less than 0.20. However, above this critical volume fraction a second series of events occurs (see Stage 2). Now the growing hybrid nanoparticles interact with one (or more) of the smaller, solvent-swollen spheres and undergo fusion and internal rearrangement to form weakly anisotropic intermediate species. The surfactant-like individual copolymer chains then adsorb onto and interact with these relatively unstable intermediates, which undergo fission to form at least two hybrid spheres of intermediate size. This hypothesis is consistent with the observations made during the *in situ* SAXS experiment (Fig. 4 and 5). In particular, the initial increase in scattered X-ray intensity at *q* = 0.019 nm^−1^ for the first 3.2 min corresponds to Stage 1, since the low *q* gradient remains close to zero (indicating that only spheres are present). However, the scattered X-ray intensity then increases significantly with a concomitant reduction in the low *q* gradient to −0.55 being observed at 7.0 min. This indicates the formation of relatively large, weakly anisotropic intermediates. Such species can be obtained as a final morphology if the volume fraction of smaller PLMA_39_–PBzMA_97_ nanoparticles lies between 0.20 and 0.35. However, at higher volume fractions, these smaller nanoparticles provide a sufficient quantity of surface-active individual copolymer chains to interact with these anisotropic intermediates. This causes the latter species to undergo fission, which produces the final hybrid spheres of intermediate diameter. This hypothesis is consistent with a prior study by Chambon and co-workers,^62^ who found that diblock copolymer vesicles prepared *via* aqueous PISA could be disrupted to form much smaller spheres when exposed to an ionic surfactant. This fission event is responsible for the gradual reduction in the scattered X-ray intensity and the low *q* gradient shown in Fig. 5.

Revisiting the representative TEM images shown in Fig. 3, we calculate (see ESI† for further details) that approximately eleven small (21 ± 2 nm) PLMA_39_–PBzMA_97_ spherical nanoparticles interact with each large (48 ± 5 nm) PLMA_39_–PBzMA_294_ sphere to form approximately four hybrid nanoparticles of 36 nm diameter. This calculation should be borne in mind when considering Scheme 3 (*i.e. n* = 11, *m* = 1 and *p* ∼ 4 for the thermal annealing experiment described in Fig. 3). However, this rather rudimentary analysis suffers from poor statistics. Thus, we also reexamined the corresponding SAXS data obtained for the thermally-annealed nanoparticles. This enabled us to calculate a fusion ratio that was remarkably close to that obtained from the above TEM image analysis (*n* ∼ 9, *m* = 1 and *p* ∼ 4), see Table S6 in the ESI.†

The driving force for the fusion process is likely to be the (partial) solvation of the nanoparticle cores by the ingress of hot solvent at elevated temperature. This inevitably leads to enhanced copolymer chain mobility, which in turn enhances the probability of micelle fusion. Indeed, we have recently published NMR evidence for such core solvation for a related PISA formulation in non-polar media.^17^ We suggest that using a higher annealing temperature (>150 °C) is likely to affect the nanoparticle hybridization mechanism. Under such conditions, the PLMA_39_–PBzMA_294_ nanoparticles may also undergo dissociation to form molecularly-dissolved copolymer chains. Clearly, this hypothesis warrants further studies. Finally, one reviewer of this manuscript has suggested that the micelle fusion/fission mechanism demonstrated herein is unlikely to apply to nanoparticles (micelles) of equal size. This is because there is no difference in free energy in this case. This may explain why a copolymer chain exchange mechanism appears to operate in such instances.^41^

## Conclusions

RAFT-mediated PISA was used to prepare sterically-stabilized PLMA_39_–PBzMA_97_ and PLMA_39_–PBzMA_294_ diblock copolymer spheres in *n*-dodecane, with mean core diameters of 21 ± 2 nm and 48 ± 5 nm, respectively. Variable-temperature SAXS analysis of these dilute dispersions at 150 °C indicates substantial core solvation and a significant reduction in aggregation number for the smaller PLMA_39_–PBzMA_97_ spheres. In contrast, changes were much less pronounced for the larger PLMA_39_–PBzMA_294_ spheres: after returning to 25 °C, approximately the same core diameters were observed before and after thermal annealing.

Annealing an equivolume *binary mixture* of these two dispersions at 1.0 % w/w led to the formation of hybrid nanoparticles with an *intermediate* mean core diameter of 36 ± 3 nm. This suggests that the copolymer chains expelled from the PLMA_39_–PBzMA_97_ spheres exhibit surfactant-like behavior and cause fission of the larger PLMA_39_–PBzMA_294_ spheres. Moreover, *in situ* SAXS studies during such experiments revealed an upturn in the low *q* gradient. This indicates the presence of weakly anisotropic transient species, which then induce fusion to form the final hybrid spherical nanoparticles of intermediate size.

Further insights regarding the nanoparticle hybridization mechanism were obtained by annealing a series of binary mixtures with varying proportions of PLMA_39_–PBzMA_97_ and PLMA_39_–PBzMA_294_ spheres at 1.0% w/w solids. Spherical nanoparticles of intermediate size were produced for PLMA_39_–PBzMA_97_ volume fractions of between 0.40 and 0.50. Interestingly, using volume fractions between 0.05 and 0.20 yielded spherical nanoparticles with slightly larger mean particle diameters than the original PLMA_39_–PBzMA_294_ spheres. This suggests that the hybridization mechanism involves initial expulsion of copolymer chains from the small PLMA_39_–PBzMA_97_ spheres and their subsequent incorporation within the larger PLMA_39_–PBzMA_294_ spheres. Moreover, TEM and SAXS analyses indicate that weakly anisotropic nanoparticles can be obtained as a final (mixed) copolymer morphology over a restricted range of compositions (*e.g.* volume fractions of 0.20–0.35 for the smaller PLMA_39_–PBzMA_97_ spheres) when heating such binary mixtures of spheres up to 150 °C. As far as we are aware, such non-isotropic species have not been observed for any nanoparticle hybridization experiments reported in the literature. Finally, we provide the first compelling experimental evidence for a micelle fusion–fission mechanism in such systems. A two-stage mechanism that involves both expulsion/insertion and micelle fusion/fission events is proposed to account for our observations.

## Conflicts of interest

There are no conflicts to declare.

## Supplementary Material

SC-011-D0SC00569J-s001
