# Supplementary material for: SAXS studies of the thermally-induced fusion of diblock copolymer spheres: formation of hybrid nanoparticles of intermediate size and shape
Source: Chem Sci. 2020 Mar 27;11(17):4312–21. doi: 10.1039/d0sc00569j (PMC8152590; doi:10.1039/d0sc00569j)
Supplement: SC-011-D0SC00569J-s001 [file SC-011-D0SC00569J-s001.pdf]

Electronic Supplementary Material (ESI) for Chemical Science.
This journal is © The Royal Society of Chemistry 2020

Supporting Information for:

SAXS Studies of the Thermally-Induced Fusion of Diblock Copolymer Spheres: Formation of Hybrid Nanoparticles of Intermediate Size and Shape

E. J. Cornel,[1] P. S. O'Hora,[2] T. Smith,[2] D. J. Growney,[2] O. O. Mykhaylyk[1,] and S. P. Armes[1,]**

1. Dainton Building, Department of Chemistry, University of Sheffield, Brook Hill, Sheffield, South Yorkshire S3 7HF, UK.
2. Lubrizol Ltd, Nether Lane, Hazelwood, Derbyshire, DE56 4AN, UK.

Authors to whom correspondence should be addressed (o.mykhaylyk@sheffield.ac.uk and s.p.armes@sheffield.ac.uk).

Contents:

1. Materials and methods (for synthesis and characterization) **S2–S5**

2. Small-angle X-ray scattering (SAXS) models and parameters **S6–S9**

3. SAXS patterns and fitting results **S10–S14**

4. Conservation of mass calculation **S15-S16**

5. References **S17**

1. Experimental

Materials. Lauryl methacrylate and benzyl methacrylate were purchased from Sigma Aldrich (UK) and each passed through a basic alumina column prior to use. *Tert*-Butyl peroxy-2-ethylhexanoate (Trigonox 21S or T21s) initiator was supplied by AkzoNobel (The Netherlands). *n*-Dodecane, THF, triethylamine, butylated hydroxytoluene and 2,2-azobis(2-methylpropionitrile) (AIBN) were purchased from Sigma-Aldrich (UK). CD_2Cl_2 was obtained from Cambridge Isotope Laboratory (USA), while $CDCl_3$ was purchased from VWR (UK). 4-Cyano-4-((2-phenylethanesulfonyl)thiocarbonylsulfanyl)pentanoic acid (PETTC) was prepared in-house according to a well-established protocol.[1]

Methods

PLMA Macro-CTA Synthesis via RAFT Solution Polymerization A near-monodisperse $PLMA_{39}$ macro-CTA was prepared via RAFT solution polymerization of LMA (59.95 g, 235.6 mmol) at 70 °C in 62.0 g toluene using PETTC (2.00 g, 5.89 mmol; target DP = 40) and AIBN initiator (0.19 g, 1.18 mmol; PETTC/AIBN molar ratio = 5.0). This polymerization was quenched after 4 h (76% conversion) to avoid loss of RAFT chain-ends under monomer-starved conditions. A mean DP of 39 was determined by ^1H NMR in CD_2Cl_2 via end-group analysis by comparing the integral of the aromatic PETTC signals at 7.1–8.1 ppm to that of the oxymethylene signals at 3.7–4.2 ppm assigned to the LMA repeat units. GPC analysis indicated a M_n of 9700 and an M_w/M_n of 1.12, which is consistent with previous studies of well-controlled RAFT polymerizations.[2-4]

Synthesis of Spherical $PLMA_{39}$-$PBzMA_x$ Diblock Copolymer Nanoparticles via RAFT Dispersion Polymerization Benzyl methacrylate (BzMA; 0.33 ml 0.34 g, 1.95 mmol) was used for the chain extension of a $PLMA_{39}$ macro-CTA (0.20 g, 0.019 mmol; target DP = 100) in 2.17 g *n*-dodecane at 20% w/w solids, using T21s initiator (16 µl of a 10% v/v solution; macro-CTA/initiator molar ratio = 3.0). This reaction mixture was heated to 90 °C for 16 h and a final BzMA conversion of 97% was determined by ^1H NMR analysis of the crude reaction mixture diluted in $CDCl_3$ (to ensure dissolution of the diblock copolymer nanoparticles), see **Figure S1** and **Equation S1**. The integrated BzMA monomer vinyl signals at 5.59 and 6.17 ppm were compared to that of the integrated benzyl proton signal assigned to the polymerized BzMA repeat units at 4.77-5.00 ppm). A PBzMA DP of 300 was targeted at 20% w/w solids using

$PLMA_{39}$ macro-CTA (0.10 g, 0.01 mmol), BzMA (0.50 ml, 0.52 g, 1.70 mmol) of BzMA, *n*-dodecane (2.46 g) and 8 µl of a 10% v/v solution of T21S. A final conversion of 98% was obtained, yielding a final PBzMA core-forming block DP of 294.

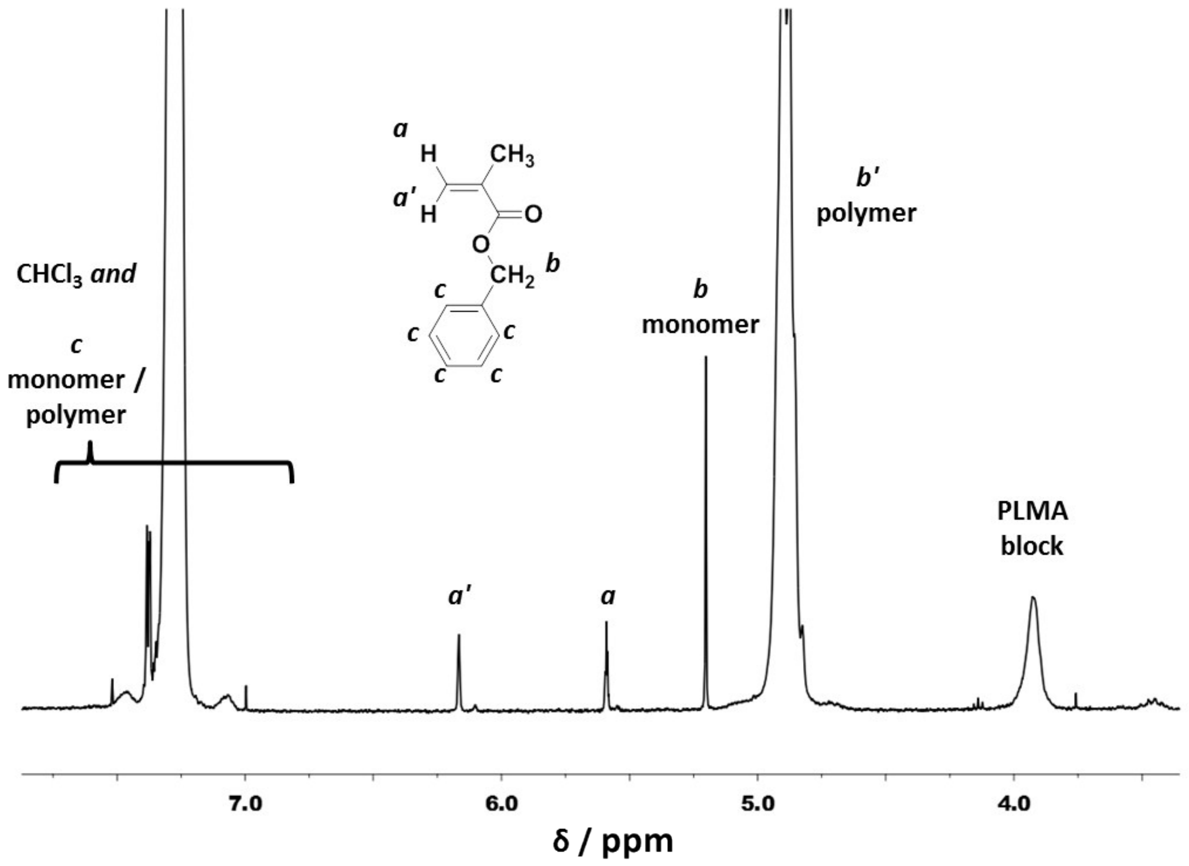

Figure S1. Representative proton NMR spectrum recorded for the crude reaction mixture of $PLMA_{39}$-$PBzMA_{294}$ nanoparticles, dissolved in $CDCl_3$ prior to analysis. Monomer conversions were calculated according to **Equation S1**, using the integrated monomer vinyl signals (*a* and *a'*) and the integrated monomer and polymer benzyl signals ($b_{monomer}$ and $b'_{polymer}$).

$$BzMA\ conversion\ (\%) = \left[1 - \frac{(a + a')}{(b_{monomer} + b'_{polymer})}\right] \times 100\% \quad (S1)$$

Characterization methods

THF GPC. Molecular weight distributions were assessed by gel permeation chromatography (GPC) using THF as an eluent. The GPC set-up comprised an Agilent 1260 Infinity series degasser and pump, two Agilent PLgel 5 µm Mixed-C columns in series and a refractive index detector. The mobile phase contained 2.0% v/v trimethylamine and 0.05% w/w butylhydroxytoluene (BHT) and the flow rate was 1.0 ml min^{-1}. Samples were dissolved in THF containing 0.50% v/v toluene as a flow rate marker prior to GPC analysis. A series of ten near-monodisperse poly(methyl methacrylate) standards (M_p values ranging from 1 280 to 330 000 g mol^{-1}) were used for calibration.

NMR spectroscopy. Proton NMR spectra were recorded in either $CDCl_3$ or CD_2Cl_2 using a Bruker AV3HD-400 or 500 MHz spectrometer. Spectra were analyzed using TopSpin version 3.1 software. For the $PLMA_{39}$-$PBzMA_x$ synthesis, the final BzMA monomer conversion was determined by dissolution of a small volume of the crude dispersion in $CDCl_3$ prior to analysis.

Transmission Electron Microscopy (TEM). A positive TEM stain was prepared by dissolving ruthenium(IV) oxide hydrate (0.30 g) and sodium periodate (2.00 g) in 50 ml water. Copolymer dispersions were diluted to 0.02% w/w using *n*-dodecane and dispersed on carbon-coated copper TEM grids. Loaded grids were stained for 7 min by exposure to aqueous solution of the TEM stain in a desiccator. TEM images were recorded using a Tecnai Spirit T12 TEM instrument operating at 80 kV and equipped with an Orius SC1000B CCD camera.

Small-angle X-ray scattering (SAXS). Experiments were performed at the ESRF (Grenoble, France) using the ID02 beamline. A monochromatic X-ray beam (wavelength (λ) = 0.0995 nm) and a 2D SAXS detector (Rayonix MX-170HS) were used for these studies. A q range of 0.012–1.50 nm^{-1} was employed for measurements, where $q = (4\pi \sin \theta)/\lambda$ corresponds to the modulus of the scattering vector and θ is half of the scattering angle. For time-resolved measurements, a glass capillary of 2 mm thickness was inserted into a heating stage (HFSX350-CAP, Linkam Scientific Instruments, Tadworth, UK). X-ray scattering data were reduced (calibration, integration and background subtraction) using standard routines available at the ID02 beamline. A scattering intensity of water was used for the absolute scale calibration of the X-ray scattering patterns. Data were fitted using either a spherical micelle model[5-7] or the Debye function[8] for the molecularly-dissolved $PLMA_{39}$ chains using the Irena package[9] for Igor Pro.

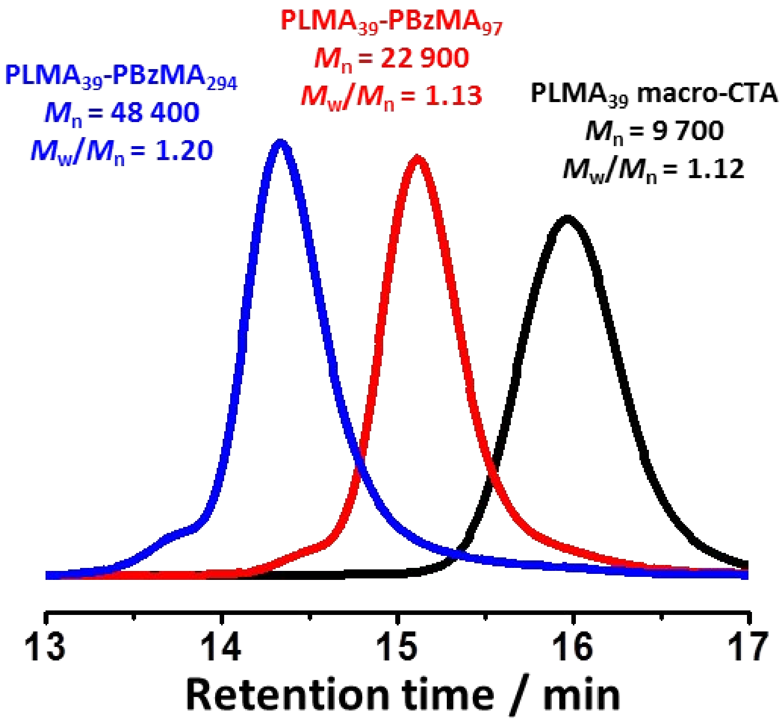

Figure S2. Normalized THF GPC traces recorded using a refractive index detector for a $PLMA_{39}$ macro-CTA, and corresponding $PLMA_{39}$-$PBzMA_{97}$ and $PLMA_{39}$-$PBzMA_{294}$ diblock copolymers. Unimodal molecular weight distributions were obtained, indicating good control over the RAFT dispersion polymerization of BzMA. A clear shift towards lower retention times indicates high blocking efficiencies.

2. Small-angle X-ray Scattering (SAXS) models and parameters

SAXS model for molecularly-dissolved $PLMA_{39}$ chains in *n*-dodecane

In order to determine the mean radius of gyration of the $PLMA_{39}$ polymer coil (R_g), a SAXS pattern recorded for molecularly-dissolved $PLMA_{39}$ chains (or unimers) in *n*-dodecane was fitted using the form factor for Gaussian chains expressed via the Debye function[8]:

$$F_c(q,R_g) = \frac{2\left[\exp\left(-q^2{R_g}^2\right) - 1 + q^2{R_g}^2\right]}{q^4{R_g}^4} \quad (S2)$$

SAXS model for dilute $PLMA_{39}$–$PMMA_x$ and $PLMA_{39}$–d_8PMMA_x spheres

In general, the differential scattering cross-section per unit sample volume $\frac{d\Sigma}{d\Omega}(q)$ for a dispersion of particles can be expressed as:

$$\frac{d\Sigma}{d\Omega}(q) = NS_{SF}(q)\int_0^\infty ... \int_0^\infty F(q,r_1,...,r_k)\Psi(r_1,...,r_k)dr_1...dr_k \quad (S3)$$

where $S_{SF}(q)$ is the structure factor. This factor describes variations of scattering from randomly arranged particles, which become more pronounced when considering either strongly interacting scattering objects or densely-packed concentrated dispersions. Since only dilute dispersions are studied, it was assumed in the SAXS data analysis that $S_{SF}(q) = 1$. $F(q,r_1,...,r_k)$ is the particle form factor expressed in terms of a set of k parameters , $\Psi(r_1,...,r_k)$ is the parameter distribution function and *N* is the particle number density per unit sample volume, which is generally expressed as:

$$N = \frac{\varphi}{\int_0^\infty ... \int_0^\infty V(r_1,...,r_k)\Psi(r_1,...,r_k)dr_1...dr_k} \quad (S4)$$

where $V(r_1,...,r_k)$ is the particle volume and φ is the particle volume fraction in the dispersion. For spherical micelles it is sufficient to consider only polydispersity of the micelle core radius, defined as r_1, usually expressed as a Gaussian distribution:

$$\Psi(r_1) = \frac{1}{\sqrt{2\pi{\sigma_{Rs}}^2}}exp\left(-\frac{(r_1 - R_s)^2}{2{\sigma_{Rs}}^2}\right) \quad (S5)$$

where R_s is the mean micelle core radius, σ_{Rs} is its standard deviation and σ_{Rs}/R_s represents the polydispersity. All other fitting parameters describing the micelle structural model for the sake of

simplicity could be considered monodisperse (their distribution functions correspond to Dirac's delta function).

The particle form factor (**Equation S3**) for spherical micelles can be expressed as:[10]

$$F(q,r_1) = N_s^2(r_1)\beta_s^2 A_s^2(q,r_1) + N_s(r_1)\beta_c^2 F_c(q,R_g) + N_s(r_1)[N_s(r_1 \text{[illegible]} \; 2N_s^2(r_1)\beta_s\beta_c A_s(q,r_1)A_c(q,r_1) \quad (S6)$$

where $N_s(r_1)$ is the volume-average aggregation number (or total number of copolymer chains per spherical nanoparticle):

$$N_s(r_1) = (1 - X_{sol})\frac{\frac{4}{3}\pi r_1^3}{V_s} \quad (S7)$$

where X_{sol} is the fraction of solvent in the micelle core and V_s is the volume of a single core-forming block (**Table S1**). Here N_{agg} is defined such that $N_s = N_{agg}$ when $r_1 = R$ [see equation (1) in the main manuscript]. β_s and β_c represent the total excess scattering length of the core-forming block (PBzMA) and the corona-forming block (PLMA), respectively. These values can be calculated by $\beta_s = V_s(\xi_s - \xi_{solv})$ and $\beta_c = V_c(\xi_c - \xi_{solv})$, where V_c is the volume of a single corona-forming block (**Table S1**); ξ_s, ξ_c and ξ_{solv} are the scattering length densities (SLDs) of the core, corona and solvent (see **Table S1** for the calculated X-ray SLDs of each component). Scattering amplitude of the spherical core in **Equation S6** is expressed as:

$$A_s(q,r_1) = \Phi(qr_1)exp\left(-\frac{q^2\sigma^2}{2}\right) \quad (S8)$$

where

$$\Phi(qr_1) = \frac{3[\sin(qr_1) - qr_1 cos(qr_1)]}{(qr_1)^3} \quad (S9)$$

A sigmoidal interface between the micelle core and corona was assumed in **Equation S8**. This is described by the exponent term with a width σ accounting for a decaying scattering length density at the membrane surface. This σ value was fixed at 0.22 nm during fitting. The self-correlation term for the corona block, $F_c(q)$, is given by the Debye function (**Equation S2**). The scattering amplitude of the corona self-term in **Equation S6** was obtained from a normalized Fourier transform of the radial density distribution function of the coronal chains in the micelles:

$$A_c(q,r_1) = \frac{\int_{r_1}^{r_1+2s} \mu_c(r)\frac{\sin(qr)}{qr}r^2dr}{\int_{r_1}^{r_1+2s} \mu_c(r)r^2dr} exp\left(-\frac{q^2\sigma^2}{2}\right) \quad (S10)$$

The radial profile of the density distribution, $\mu_c(r)$, is expressed by a linear combination of two cubic b splines, with two fitting parameters *s* and *a* corresponding to the width of the scattering length density corona profile and the function weight coefficient, respectively. This information can be found elsewhere,[11,12] as can the approximate integrated form of **Equation S10**.

According to **Equation S4** the number density per unit sample volume for the spherical micelles can be expressed as:

$$N = \frac{\varphi}{\int_0^\infty V(r_1)\Psi(r_1)dr_1} \quad (S11)$$

where, $V(r_1)$ is the total volume of copolymer in a spherical micelle $[V(r_1) = (V_s + V_c)N_s(r_1)]$.

SAXS model parameters (copolymer block volumes and SLDs)

The volumes of each copolymer block, *i.e.* $PLMA_{39}$ and $PBzMA_x$ (**Table S1**) were calculated using

$$V_b = DP \cdot v_m \quad (S12)$$

where DP is the degree of polymerization and v_m is the volume of the repeat unit in each block such that $v_m = \frac{M_{Mon}}{N_A\rho}$, where M_{Mon} is the molecular weight of the corresponding block repeat unit, N_A is Avogadro's number and ρ is the mass density of either the core-forming or corona-forming block.

X-ray scattering length densities were calculated for each component of the nanoparticle dispersion (**Table S1**) using:

$$\xi = \frac{\sum_{i=1}^{L} n_i b_i}{v_m} \quad (S13)$$

where L is the number of types of chemical elements in a compound, n_i is the number of atoms of the ith chemical element, b_i is the scattering length of the ith chemical element atom (determined by its number of electrons multiplied by scattering length of an electron), and v_m is the molecular volume of either a single monomer repeat unit or a solvent molecule.

Table S1. Summary of mean block volumes, mass densities and X-ray SLDs used for SAXS modeling. Mean block volumes and SLDs were calculated for a range of temperatures assuming that the thermal expansion coefficients of PLMA and PBzMA were equal to that of reported by Fetters and co-workers for poly(*n*-butyl methacrylate) and polystyrene, respectively.[13]

Polymer block	Temperature / °C	Block volume / Å^3	Mass density / g cm^{-3}	X-ray SLD (× 10^{-6}) / Å^{-2}
$PLMA_{39}$	25	17 766	0.927	8.780
	50	18 021	0.914	8.657
	75	18 283	0.901	8.534
	100	18 553	0.888	8.411
	125	18 830	0.875	8.288
	150	19 117	0.862	8.165
$PBzMA_{294}$	25	73 127	1.176	10.65
	50	73 951	1.163	10.53
	75	74 794	1.150	10.41
	100	75 656	1.137	10.29
	125	76 538	1.124	10.17
	150	77 441	1.111	10.06
$PBzMA_{97}$	25	24 127	1.176	10.65
	50	24 399	1.163	10.53
	75	24 677	1.150	10.41
	100	24 961	1.137	10.29
	125	25 252	1.124	10.17
	150	25 550	1.111	10.06
n-Dodecane	25	-	0.746	7.283
	50	-	0.727	7.098
	75	-	0.709	6.922
	100	-	0.690	6.737

	125	-	0.671	6.551
	150	-	0.651	6.356

3. SAXS Analysis Results

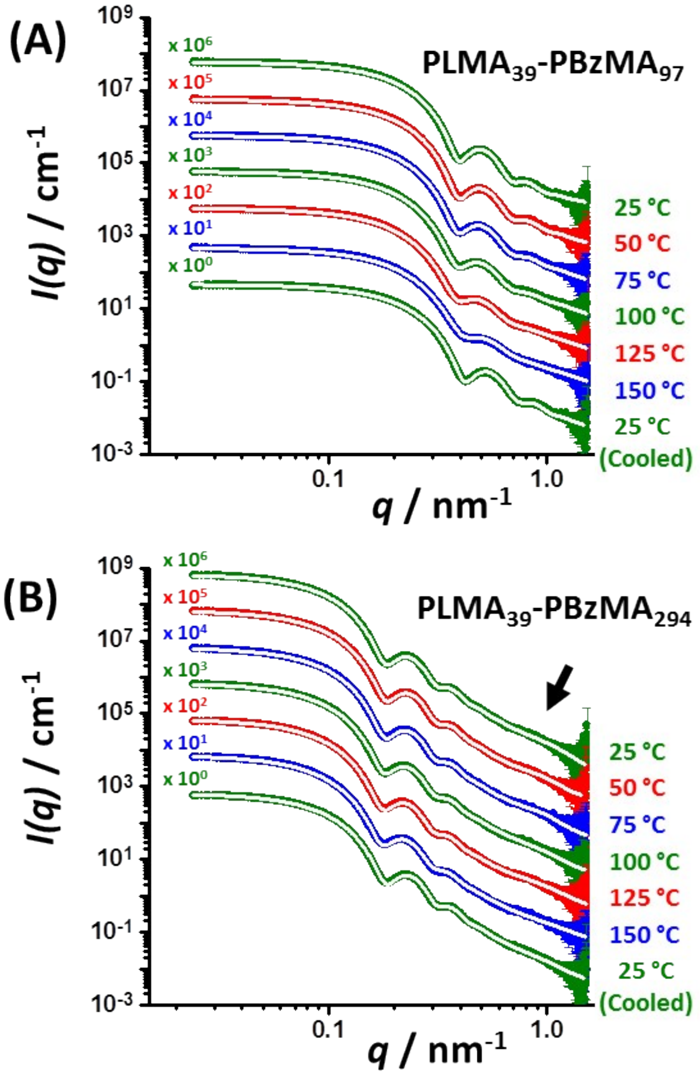

Figure S3. SAXS patterns recorded for (A) $PLMA_{39}$-$PBzMA_{97}$ and (B) $PLMA_{39}$-$PBzMA_{294}$ spheres at 1.0% w/w in *n*-dodecane at various temperatures. Heating the latter nanoparticles up to 150 °C caused minimal change in their scattering pattern. Differences between the scattering patterns recorded at 25 °C and 150 °C for the smaller $PLMA_{39}$-$PBzMA_{97}$ spheres indicate significant core solvation and partial nanoparticle dissociation to form molecularly-dissolved copolymer chains. Annealed dispersions were cooled to 25 °C before collecting the final scattering pattern. The black arrow indicates a broad diffuse peak which appears to correspond to the radius of gyration of the PBzMA chains (~4 nm) within the nanoparticle cores. White traces indicate data fits obtained using a spherical micelle model.[5,7]

Table S2. SAXS data fits recorded for $PLMA_{39}$-$PBzMA_{97}$ spherical nanoparticles at various temperatures from 25 °C to 150 °C. $PLMA_{39}$ and $PBzMA_{97}$ core volumes were calculated for each temperature by assuming that the thermal expansion coefficients of PLMA and PBzMA were equal to that reported by Fetters and co-workers for poly(*n*-butyl methacrylate) and polystyrene, respectively.[13] In order to account for the nanoparticle dissociation during thermal annealing, an additional population for the molecularly-dissolved copolymer chains, represented by the Debye function (**Equation S2**), has been added to the SAXS intensity equation used to fit these patterns. The radius of gyration for these copolymer chains was calculated to be 2.97 nm using $R_g = [(DP \times 0.255 \times 1.53)/6]^{0.5}$ where DP = 39 + 97, and "DP x 0.255" and "1.53" correspond to the contour length and Kuhn length of the copolymer chains, respectively. This approach assumes that both blocks have the same repeat unit length and that their Kuhn length is equal to that of poly(methyl methacrylate). [13]

**Temperature / °C**	**Conc. Spheres % v/v**	**Conc. Chains % v/v**	**Core diameter / nm**	**Polydispersity (σ_{Rs}/R_s)**	**Shell R_g / nm**	**Core solvation / %**
25[a]	0.67	0.00	21.1 ± 1.7	0.08	2.0	0.00
50	0.60	0.04	21.2 ± 1.7	0.08	2.0	0.06
75	0.57	0.06	21.4 ± 1.8	0.08	2.0	0.11
100	0.55	0.08	21.5 ± 2.0	0.09	2.0	0.14
125	0.52	0.10	21.5 ± 2.3	0.11	2.0	0.16
150	0.45	0.14	20.4 ± 2.4	0.12	2.0	0.18
25[b]	0.60	0.06	19.9 ± 1.6	0.08	2.0	0.00

[a]As prepared via RAFT-mediated PISA.
[b]After cooling from 150 °C to 25 °C.

Table S3. SAXS data fits recorded for $PLMA_{39}$-$PBzMA_{294}$ spherical nanoparticles at various temperatures from 25 °C to 150 °C. The same approach as that described in the **Table S2** caption was used for the SAXS data fit. The radius of gyration of the molecularly-dissolved copolymer chains was calculated to be R_g = 4.65 nm. In addition, a Gaussian function was included in the SAXS intensity equation to account for the broad diffuse peak observed at high *q* (**Figure S3B**). The mean peak position for this feature is at *q* = 0.74 nm and its full width at half maximum is 0.32 nm.

**Temperature / °C**	**Conc. Spheres % v/v**	**Conc. Chains % v/v**	**Core diameter / nm**	**Polydispersity (σ_{Rs}/R_s)**	**Shell R_g / nm**	**Core solvation / %**
25[a]	0.78	0.00	48.1 ± 4.9	0.10	2.0	0.00
50	0.75	0.00	48.1 ± 4.9	0.10	2.0	0.00
75	0.76	0.07	48.5 ± 4.9	0.10	2.0	0.00
100	0.74	0.07	49.0 ± 5.0	0.10	2.0	0.00
125	0.73	0.09	49.5 ± 5.1	0.10	2.0	0.00
150	0.73	0.11	49.9 ± 5.3	0.11	2.0	0.00
25[b]	0.76	0.00	48.1 ± 4.8	0.10	2.0	0.00

[a]As prepared via RAFT-mediated PISA.
[b]After cooling from 150 °C to 25 °C.

The SAXS pattern recorded for the $PLMA_{39}$ macro CTA (**Figure S4**) was fitted using the Debye function (**Equation S2).** The spherical micelle model (**Equations S3-S13**) was used for analyzing scattering patterns obtained for dilute copolymer dispersions of the $PLMA_{39}$–$PBzMA_x$ spheres. The mean volumes and SLDs calculated for each block (**Table S1**) were fixed during fitting. Geometric parameters for the micelles (*i.e.* the R_g of the micelle corona block, and the mean core radius and its standard deviation) and the copolymer volume fraction were allowed to vary until the best SAXS data fits were obtained (**Tables S2 and S3**). Mean volumes for the core-forming PBzMA block were calculated based on the corresponding mixing ratio when fitting the SAXS data recorded for nanoparticles of intermediate size that are formed after thermally-induced hybridization.

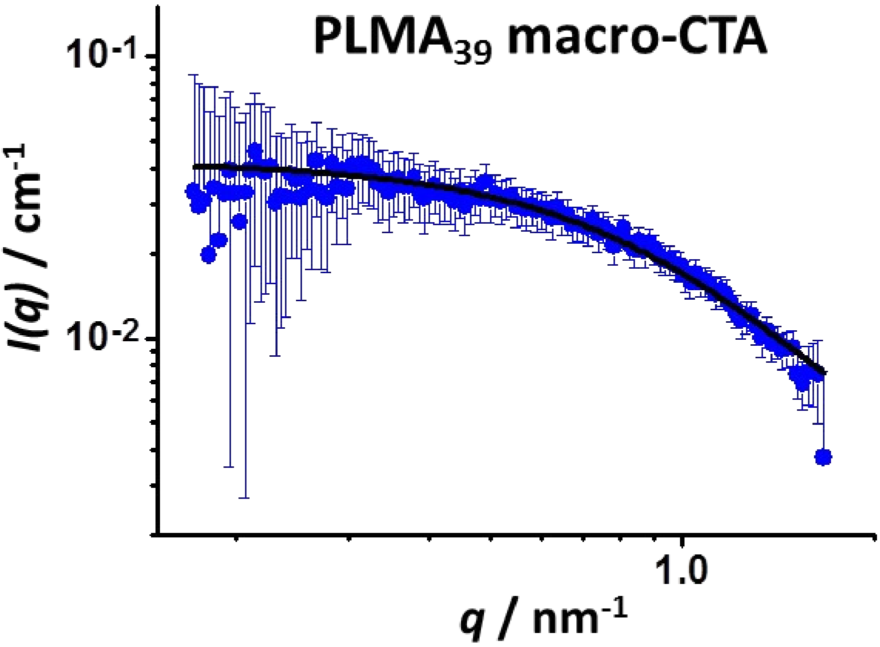

Figure S4. SAXS pattern recorded for a 1.0% w/w solution of the $PLMA_{39}$ precursor molecularly dissolved in *n*-dodecane. The black line corresponds to the data fit obtained using the Debye function[8] (**Equation S2**). This analysis indicated a mean radius of gyration, R_g, of 1.91 nm.

Table S4. Summary of SAXS fitting parameters for the initial $PLMA_{39}$-$PBzMA_{97}$ and $PLMA_{39}$-$PBzMA_{294}$ binary particle dispersions at 25 °C, an equivolume binary dispersion of these two types of nanoparticles before annealing and the hybrid nanoparticles formed after the annealing this binary mixture at 150 °C for 1 h. The latter two entries were both analyzed at 20 °C.

**Formulation**	**Core-forming block volume / Å³**	**Concentration / % v/v**	**$PBzMA_x$ core diameter / nm**	**Polydispersity (σ_{Rs}/R_s)**	**$PLMA_{39}$ shell R_g / nm**
$PLMA_{39}$-$PBzMA_{294}$	73 127	0.78	48.1 ± 4.9	0.10	2.0
$PLMA_{39}$-$PBzMA_{97}$	24 127	0.67	21.1 ± 1.7	0.08	2.0
Binary mixture (1:1 v/v ratio)	73 127 / 24 127	0.22 / 0.22	48.1 ± 4.9 / 21.1 ± 1.7	0.10 / 0.08	2.0 / 2.0
Hybrid nanoparticles*	52260	0.48	35.8 ± 3.5	0.10	2.0

*This pattern was fitted using a Debye function to account for a population of molecularly-dissolved diblock copolymer chains (concentration = 0.02 v/v, R_g = 2.97 nm). In addition, a diffraction peak was included in the data fit (its peak position is at q = 0.72 nm and its full width at half maximum is 0.40 nm).

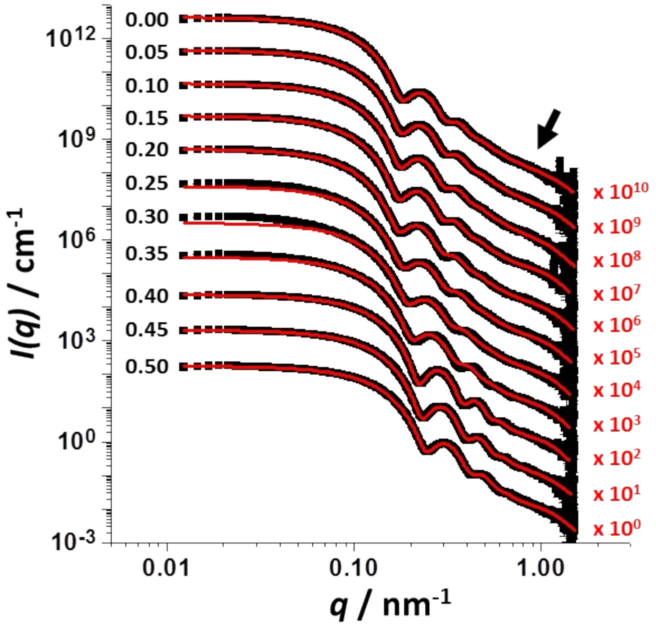

Figure S5. SAXS scattering patterns recorded at 20 °C for 1.0% w/w binary mixtures of $PLMA_{39}$-$PBzMA_{97}$ and $PLMA_{39}$-$PBzMA_{294}$ spheres after thermal annealing at 150 °C for 1 h. Caption labels denote the relative volume fraction of the $PLMA_{39}$-$PBzMA_{97}$ spheres used in these experiments. Red lines represent fits of the spherical micelle model to the experimental SAXS data. Unsatisfactory data fits were obtained for relative volume fractions of 0.25, 0.30 and 0.35, suggesting weak anisotropic character for such hybrid nanoparticles. This is consistent with the TEM images (see **Figure 6** in the main manuscript). Each pattern is offset by an arbitrary numerical factor to aid clarity. A diffraction peak was included in the fit to account for the broad diffuse peak at high q (this feature is located at q = 0.80 nm – see black arrow - and its full width at half maximum is 0.38 nm).

Table S5. Summary of SAXS fitting parameters obtained for $PLMA_{39}$-$PBzMA_{97}$ and $PLMA_{39}$-$PBzMA_{294}$ dispersions and the hybrid nanoparticles formed by annealing such binary mixtures at 150 °C for 1 h (**Figure S5**). Mean volumes for the core block were calculated from the initial core volumes and nanoparticle mixing ratios by assuming full entropic mixing during the hybridization experiments. All data were fitted by assuming no solvent within the nanoparticle cores and a fixed shell volume of 17716 Å^3. A diffraction peak was included in the fit to account for the broad diffuse peak at high q (this feature is located at q = 0.80 nm and its full width at half maximum is 0.38 nm).

**$PBzMAB_{97}$ / $PBzMA_{294}$ volume ratio**	**Average core volume / Å^3**	**Concentration / % v/v**	**$PBzMA_x$ core diameter / nm**	**Polydispersity (σ_{Rs}/R_s)**	**$PLMA_{39}$ shell R_g / nm**
0 / 100	72222	0.51	48.4 ± 4.9	0.10	2.0
5 / 95	70413	0.54	48.8 ±4.8	0.10	1.9
10 / 90	68556	0.53	49.4 ±4.8	0.10	2.0
15 / 85	66649	0.56	50.1 ± 4.9	0.10	2.0
20 / 80	64691	0.56	50.3 ± 5.2	0.10	2.0
25 / 75	62678	0.50	47.8 ± 5.2	0.11	2.0
30 / 70	60609	0.47	45.0 ± 5.1	0.11	2.0
35 / 65	58481	0.52	42.6 ± 4.6	0.11	2.0
40 / 60	56291	0.51	39.1 ± 3.2	0.08	2.0
45 / 55	54038	0.50	37.5 ± 3.3	0.09	2.0
50 / 50	51718	0.48	35.7 ± 3.5	0.10	1.9

Conservation of Mass Calculations for Thermal Annealing Experiments

Analysis of TEM images shown in Figures 3C and 3D

One of the reviewers of this manuscript suggested that we should consider the principle of the conservation of mass when discussing fusion-fission mechanism (see Scheme 3). In this context, we must consider the number of particles per unit volume. TEM images obtained for the diluted, dried binary mixture before thermal annealing (**Figure 3C**), and the same mixture after thermal annealing (**Figure 3D**) are shown in the main manuscript (and are reproduced below for convenience).

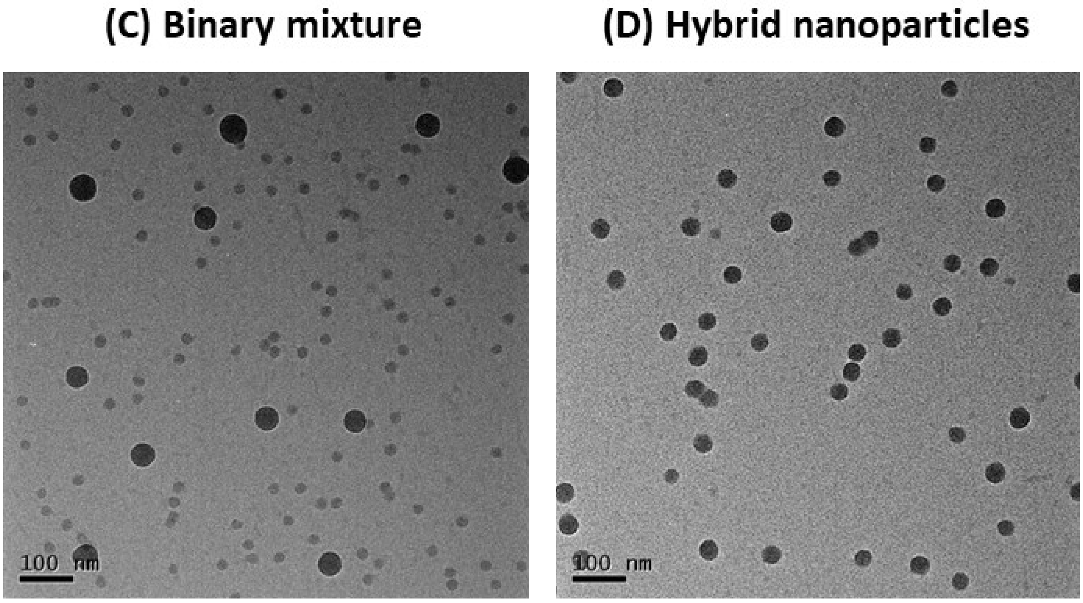

The former image contains 11 large nanoparticles of approximately 24 nm radius and 119 smaller nanoparticles with a mean radius of 10.5 nm (See above). The latter image contains 46 hybrid nanoparticles with a mean radius of 18 nm. From these images, we can estimate the mean nanoparticle volumes before and after thermal annealing:

Mean volume of small particles in the initial binary mixture = 119 x (10.6 nm)^3 = 139,753 nm^3
Mean volume of large particles in the initial binary mixture = 11 x (24.1 nm)^3 = 153,016 nm^3
Hence the total volume of the initial nanoparticles = 139,753 + 153,016 = **292,751 nm^3**

The total volume for the thermally-annealed hybrid nanoparticles = 46 x (17.9 nm)^3 = **263,826 nm^3**

Thus, the total nanoparticle volumes differ by only 11% before and after thermal annealing. Given the relatively low sampling statistics, this rather small difference is likely to be within experimental error, which suggests that the principle of conversation of mass is most likely valid.

From the above data, we can also estimate the number of nanoparticles involved in the fusion-fission event for each large nanoparticle. Thus, on average (119 ÷ 11 = 10.8) ~ 11 small nanoparticles interact with one large particle to form (46 ÷ 11 = 4.2) ~ 4 hybrid nanoparticles. This point should be borne in mind when considering **Scheme 3**.

Analysis of SAXS data shown in Figure 3

Particle size information obtained from SAXS analysis was used to determine the number of nanoparticles involved in the fusion-fission event for each large nanoparticle estimated in the preceding TEM image analysis. First, the volume of the nanoparticle cores, *V*, was calculated using *V* = $4/3 \times \pi \times R^3$, where *R* is the mean radius of the spherical PBzMA core. Thus, the total volume of PBzMA present in the dilute binary dispersion was calculated. Then the volume fraction of PBzMA present in each individual dispersion (i.e. $PLMA_{39}$-$PBzMA_{97}$ and $PLMA_{39}$-$PBzMA_{294}$) was calculated using:

$$VPBzMA = \left(\frac{BzMA\ (g) \times \left[\frac{Monomer\ Conv.}{100}\right] / Density\ of\ PBzMA\ (g/L)}{Total\ Sample\ Volume} \right) \quad (S14)$$

where the total sample volume is equal to the sum of the volumes of the BzMA monomer, $PLMA_{39}$ homopolymer precursor and solvent, respectively.

The as-synthesized dispersions of small and large spherical nanoparticles were mixed in a 1:1 v/v ratio (50 µL of each dispersion) and diluted with *n*-dodecane to 1.0 % w/w. The total volume of PBzMA within this binary dispersion was calculated by multiplying the volume fraction of PBzMA (from **Equation S14**) with the volume of each dispersion (50 µL). Thus, the total volume of PBzMA within the small and large nanoparticles present in this binary mixture at 1.0 % w/w solids is 4.14 µL + 5.58 µL = 9.72 µL (0.00414 mL + 0.00585 mL = 0.00972 mL).

The total number of the small spherical nanoparticles within the binary mixture before thermal annealing was calculated by dividing the total PBzMA volume for these nanoparticles by the mean PBzMA core volume for an individual small nanoparticle. Similarly, the total number of the large spherical nanoparticles within the binary mixture before thermal annealing was calculated by dividing the total PBzMA volume for these nanoparticles by the mean PBzMA core volume for an individual large nanoparticle. Thus the average number of small nanoparticles that undergo fusion with each large nanoparticle (**n**) was calculated (see **Table S6**).

Then the total number of *hybrid* nanoparticles in the thermally-annealed dispersion was calculated by dividing the total PBzMA volume in the dispersion by the volume of one spherical *hybrid* PBzMA core. Finally, the normalized number of hybrid nanoparticles of intermediate size (**p**) generated during fission was calculated (see **Table S6**). [N.B. By definition, the number of large nanoparticles (**m**) is unity].

Table S6. SAXS calculation of the number of small and large nanoparticles present in the equivolume binary mixture before thermal annealing and the number of hybrid nanoparticles produced after thermal annealing. This enables the relative number of nanoparticles (or nanoparticle ratio) to be determined.

Type of Nanoparticle	Nanoparticle Core / nm		Nanoparticle Core Volume		Number of Nanoparticles[1]	Nanoparticle Ratio[2]
	Diameter	Radius	nm^3	mL		
**Small**	21.1	10.6	4916	4.92×10^{-18}	8.42×10^{14}	8.8 (**n**)
**Large**	48.1	24.1	58239	5.82×10^{-17}	9.58×10^{13}	1.0 (**m**)
**Hybrid**	35.8	17.9	24012	2.40×10^{-17}	4.05×10^{14}	4.2 (**p**)

1. Number of nanoparticles present in the initial binary dispersion before thermal annealing or the number of hybrid nanoparticles in the annealed dispersion (both dispersions are 1.0 % w/w solids).
2. The letters refer to **Scheme 3** in the main manuscript.

4. References

(1) Semsarilar, M.; Ladmiral, V.; Blanazs, A.; Armes, S. P. Anionic Polyelectrolyte-Stabilized Nanoparticles via RAFT Aqueous Dispersion Polymerization, *Langmuir*, 2012, **28**, 914-922.
(2) Fielding, L. A.; Lane, J. A.; Derry, M. J.; Mykhaylyk, O. O.; Armes, S. P. Thermo-responsive Diblock Copolymer Worm Gels in Non-polar Solvents, *J. Am. Chem. Soc.*, 2014, **136**, 5790-5798.
(3) Chiefari, J.; Chong, Y. K.; Ercole, F.; Krstina, J.; Jeffery, J.; Le, T. P. T.; Mayadunne, R. T. A.; Meijs, G. F.; Moad, C. L.; Moad, G.; Rizzardo, E.; Thang, S. H. Living free-radical polymerization by reversible addition-fragmentation chain transfer: The RAFT process, *Macromolecules*, 1998, **31**, 5559-5562.
(4) Moad, G.; Rizzardo, E.; Thang, S. H. Toward living radical polymerization, *Acc. Chem. Res.*, 2008, **41**, 1133-1142.
(5) Pedersen, J. S.; Gerstenberg, M. C. Scattering Form Factor of Block Copolymer Micelles, *Macromolecules*, 1996, **29**, 1363-1365.
(6) Pedersen, J. S.; Schurtenberger, P. Scattering Functions of Semiflexible Polymers with and without Excluded Volume Effects, *Macromolecules*, 1996, **29**, 7602-7612.
(7) Pedersen, J. S. Form factors of block copolymer micelles with spherical, ellipsoidal and cylindrical cores, *J. App. Crystallogr.*, 2000, **33**, 637-640.
(8) Debye, P. Molecular-weight Determination by Light Scattering, *J. Phys. Colloid Chem.*, 1947, **51**, 18-32.
(9) Ilavsky, J.; Jemian, P. R. Irena: tool suite for modeling and analysis of small-angle scattering, *J. App. Crystallogr.*, 2009, **42**, 347-353.
(10) Pedersen, J. S. Form factors of block copolymer micelles with spherical, ellipsoidal and cylindrical cores, *Journal of Applied Crystallography*, 2000, **33**, 637-640.
(11) Pedersen, J. S.; Gerstenberg, M. C. The structure of P85 Pluronic block copolymer micelles determined by small-angle neutron scattering, *Colloids and Surfaces a-Physicochemical and Engineering Aspects*, 2003, **213**, 175-187.
(12) Pedersen, J. S.; Svaneborg, C.; Almdal, K.; Hamley, I. W.; Young, R. N. A small-angle neutron and X-ray contrast variation scattering study of the structure of block copolymer micelles: Corona shape and excluded volume interactions, *Macromolecules*, 2003, **36**, 416-433.
(13) Fetters, L. J.; Lohsey, D. J.; Colby, H. Physical Properties of Polymers Handbook; 2 ed.; Mark, J. E., Ed.; Springer New York: 2007, p 447-454.
